# The Role of NF-κB in Peritoneal Fibrosis and Adhesion in Humans and Animals: A Systematic Review

**DOI:** 10.3390/ijms27052199

**Published:** 2026-02-26

**Authors:** Tomasz Jasiński, Natalia Kozłowska, Łukasz Zdrojkowski, Andrzej Bręborowicz, Barbara Rey, Małgorzata Domino

**Affiliations:** 1 Department of Large Animal Diseases and Clinic, Institute of Veterinary Medicine, Warsaw University of Life Sciences, 02-787 Warsaw, Poland; tomasz_jasinski@sggw.edu.pl (T.J.); natalia_kozlowska@sggw.edu.pl (N.K.); lukasz_zdrojkowski@sggw.edu.pl (Ł.Z.); 2 Department of Pathophysiology, Poznan University of Medical Sciences, 61-701 Poznan, Poland; abreb@ump.edu.pl; 3 Scientific Circle of Biotechnologists KNBiotech, Warsaw University of Life Sciences, 02-787 Warsaw, Poland; s227056@sggw.edu.pl

**Keywords:** peritoneum, peritoneal dialysis, complication, abdominal adhesion, signaling pathway, comparative medicine

## Abstract

Peritoneal fibrosis is a consequence of peritoneal dialysis, initiated by an inflammatory response in the peritoneum, whereas peritoneal adhesions represent intra-abdominal post-inflammatory complications. Given that the nuclear factor kappa B (NF-κB) signaling pathway plays a central role in inflammation, this systematic review aims to compile research findings on the role of NF-κB in peritoneal fibrosis and adhesions. Following the PRISMA 2020 guidelines, literature searches were conducted in PubMed, Scopus, and Web of Knowledge. Inclusion criteria covered research articles investigating NF-κB in peritoneal fibrosis and adhesions. Selected studies were categorized based on NF-κB-mediated regulation and NF-κB-targeted therapies. To date, the role of NF-κB in peritoneal fibrosis and adhesions has been described in 39 publications: 29 on fibrosis, 9 on adhesions, and 1 addressing both conditions. NF-κB activation was reported in human and animal studies, both in vitro and in vivo, in response to stimuli such as high glucose, inflammatory cytokines, growth factor, bacteria, and irritants. This activation led to upregulation of specific inflammatory, mesothelial-to-mesenchymal transition, fibrosis, and angiogenesis markers. All 21 therapeutic studies demonstrated inhibition of NF-κB activity and downregulation of related molecular markers—15 in fibrosis and 6 in adhesions. Controlling NF-κB activity in the peritoneal mesothelium may be beneficial in managing peritoneal dialysis and preventing peritoneal post-inflammatory complications.

## 1. Introduction

Chronic kidney disease (CDK) is a major public health problem that often progresses to life-threatening end-stage renal disease (ESRD) [1,2,3]. For patients with ESRD, peritoneal dialysis (PD) represents one of the life-saving options for renal replacement therapy [1,4,5]. PD is a safe, effective, convenient, and home-based therapy [4,5]; however, repeated and prolonged use causes morphological changes in the peritoneum [6,7,8].

During PD, the peritoneal membrane is continuously exposed to PD solutions [4,5,8], mechanical stress [9,10], and infections [11] leading to peritoneal injury. On one side, routinely used PD solutions are acidic, hyperosmotic, and hyperglycemic because of their pH, electrolyte composition, and high concentration of glucose and glucose degradation products [12,13,14]. Consequently, these solutions exhibit non-physiological characteristics and low biocompatibility [12,13]. On the other side, intra-abdominal surgical interventions, such as PD catheter insertion [15,16], induce mechanical stress. On the third side, catheter insertion and maintenance may facilitate the entry of microorganisms into the peritoneal cavity, leading to infectious peritonitis. Microorganisms may also translocate into the peritoneal cavity from the intestine, which is contiguous with the peritoneal membrane, when the intestinal–peritoneal barrier is compromised or damaged [11]. These injuries promote inflammatory response in the peritoneum [8,10,17], initiating a series of cellular and molecular changes in mesothelial, mesenchymal, and endothelial cells that ultimately lead to peritoneal fibrosis [18,19]. Peritoneal fibrosis is characterized by progressive mesothelial damage [17,18,19,20,21,22,23,24,25], interstitial fibrosis [11,18,23,26,27], and neovascularization [23,28,29,30,31].

Mesothelial damage is characterized by a reduction or loss of mesothelial cells, which occurs through cell death, detachment, or mesothelial-to-mesenchymal transition (MMT) [17]. MMT is also referred to as epithelial-to-mesenchymal transition (EMT) [18], during which peritoneal mesothelial cells transdifferentiate from an epithelial phenotype into mesenchymal characteristics typical of submesothelial myofibroblasts [17,19]. During this process, normally, immobile mesothelial cells undergo cytoskeletal reorganization [20], loss of intercellular tight junctions [18] and associated cell-to-cell adhesion [20], and thereby acquire migratory capacity into the submesothelial compact zone [18,20]. This transdifferentiation can be induced by transforming growth factor β1 (TGF-β1) [21,22] and peritoneal inflammation [18,23], leading to underexpression of epithelial markers such as E-cadherin, and overexpression of mesenchymal-related protein such as vimentin and fibrosis-related proteins, including collagens, and fibronectin [23,24]. Through MMT, peritoneal mesothelial cells lose their physiological functions and instead acquire myofibroblast-like function, such as the secretion of extracellular matrix (ECM) components [18,25].

Interstitial fibrosis is characterized by the excessive production of connective tissue within the submesothelial compact zone, resulting in its thickening [11,23]. During interstitial fibrosis, local interstitial cells, such as submesothelial fibroblasts, become activated or differentiated into myofibroblasts [17,26]. Activated fibroblasts and myofibroblasts, originating from both fibroblast differentiation [17,26] and MMT [17,19], overproduce ECM components, including collagen I and fibronectin. This excessive ECM deposition leads to thickening of the submesothelial compact zone [17,26]. Fibroblast differentiation into myofibroblasts is triggered by TGF-β1 [27] and is characterized by the expression of α-smooth muscle actin (α-SMA) [17,26]. Consequently, during interstitial fibrosis, markers such as α-SMA, collagen I, and fibronectin are markedly overexpressed.

Neovascularization, including angiogenesis and vasculopathy, leads to changes in both the structure and number of blood vessels in the peritoneum [23,28]. Both angiogenesis and vasculopathy are induced by vascular endothelial growth factor (VEGF) [29], which is produced by activated vascular endothelial cells in response to TGF-β1 stimulation [30]. Increased angiogenesis results in an elevated solute transport rate and enhanced endothelial permeability [28], ultimately leading to a decline in the ultrafiltration capacity of the peritoneal membrane [31].

It can be observed that increased expression of TGF-β1, in mesenchymal [21,22], mesothelial [27], and endothelial cells [30] play a key role in translating injury signals into tissue remodeling. However, TGF-β1 is also involved in the inflammatory response in the peritoneum [32,33]. Increased secretion of proinflammatory cytokines, such as monocyte chemoattractant protein-1 (MCP-1), stimulates the migration and infiltration of monocytes/macrophages and circulating T cells [34], leading to their accumulation in the peritoneum [35]. Consequently, multiple leukocyte populations, particularly macrophages, are recruited to the injured area [36], where both infiltrating immune cells [21,37] and local interstitial cells are activated [17,26]. Activated macrophages produce more MCP-1, numerous other proinflammatory cytokines—including interleukin-1β (IL-1β), interleukin-6 (IL-6), interleukin-8 (IL-8), and tumor necrosis factor α (TNF-α) [6,21,37]—as well as TGF-β1 [32,33]. Through these mediators, macrophages contribute to the intensification of local inflammation [6,21,37], MMT [21,22], and fibroblasts differentiation into myofibroblasts [27], as well as the activation of fibroblasts [27], myofibroblasts [22,27], and vascular endothelial cells [30]. Moreover, MCP-1 can enhance collagen and TGF-β1 synthesis in fibroblasts [31] and induce calcium flux and respiratory burst in leukocytes, leading to the generation of reactive oxygen species (ROS) [37]. Thus, peritoneal inflammation—amplified by the TGF-β1 signaling pathway—promotes the local production of multiple mediators that induce peritoneal fibrosis and promote its progression.

Importantly, peritoneal fibrosis is not regulated solely by the well described TGF-β1 signaling pathway. Both MMT and interstitial fibrosis can also be mediated by TGF-β1-independent pathways, such as nuclear factor kappa B (NF-κB) signaling [38,39,40]. Furthermore, the peritoneal inflammation, characterized by macrophage and T-cell recruitment, is closely associated with the activation of several intracellular signaling pathways, including NF-κB [40,41]. In non-stimulated cells, NF-κB complexes are retained in the cytoplasm in an inactive state, as NF-κB subunits p50 and p65 (Rel A) are bound to inhibitory kappa B (IκB) proteins, forming an inactive trimer complex. Members of the IκB protein family function as key NF-κB inhibitors, which are phosphorylated by IκB kinase (IKK). In the canonical NF-κB pathway, stimulation by specific signals induces phosphorylation and ubiquitination of IκB, leading to its proteasomal degradation and the release of the p50/p65 heterodimer. The released dimer subsequently translocates into the nucleus, where it activates transcription of target genes [42,43]. NF-κB-regulated inflammatory targets include the production of chemokines and cytokines—such as MCP-1 [44], IL-6 [45], IL-1β [38], and TNF-α [46]—as well as adhesion molecules [47]. Therefore, NF-κB plays a central role in both physiological immune responses and pathological inflammation [48], is involved in the regulation of various fibrotic diseases [49,50,51], and has been proposed as a potential target for fibrotic disease therapy [52].

In addition to pathological inflammation of the peritoneum, the alteration of functional properties of the peritoneal membrane depends on which pathological process predominates in peritoneal fibrosis—interstitial fibrosis or neovascularization. When mesothelial damage coexists with pronounced interstitial fibrosis, peritoneal membrane hypopermeability results in reduced elimination of toxins from the bloodstream [23]. In contrast, when mesothelial damage is accompanied by enhanced neovascularization, peritoneal membrane hyperpermeability impairs ultrafiltration [31,53,54]. These peritoneal membrane dysfunctions lead to PD failure, which is a major contributor to treatment discontinuation [7,8,55]. In addition to peritoneal fibrosis, peritoneal injury may also result in post-inflammatory complications, including intra-abdominal adhesions [16,56,57]. Such peritoneal adhesions not only compromise PD efficiency [16,57] but may also lead to potentially fatal outcomes [57], particularly when adhesions form between the peritoneum and small intestine loops, causing abdominal pain and intestinal obstruction [58].

Therefore, the aim of this systematic review is to compile and organize research findings from existing publications on the role of NF-κB in peritoneal fibrosis and peritoneal adhesions. Following the PICOT (Population, Intervention, Comparison, Outcome, Time) framework, the research question is formulated as follows: “In humans and animals with peritoneal fibrosis and peritoneal adhesions (P), which aspects of the NF-κB signaling pathway (I), compared with controls (C), have been investigated (O) in existing publications (T).”

## 2. Materials and Methods

### 2.1. Eligibility and Exclusion Criteria

The inclusion criterion research articles on NF-κB in peritoneal fibrosis and NF-κB peritoneal adhesions from 1993 to November 2025. Articles without available abstract and full text availability in English were excluded.

### 2.2. Search Strategies

Literature searches were conducted in November 2025 using the following electronic search databases: PUBMED (search date: 17 November 2025; search strategy: #1: “peritoneal fibrosis”[tw] OR “peritoneal adhesion*”[tw], #2: NF-κB[tw] OR “nuclear factor κB”[tw] OR “nuclear factor kappa B”[tw], #3: #1 AND #2; retrieved records: 27); Scopus (search date: 17 November 2025; search strategy: TITLE-ABS-KEY (“peritoneal fibrosis” OR “peritoneal adhesion*”) AND (NF-κB OR “nuclear factor κB” OR “nuclear factor kappa B”); retrieved records: 243); Web of Knowledge (search date: 17 November 2025; search strategy: #1: TS = (“peritoneal fibrosis” OR “peritoneal adhesion*”), #2: TS = (NF-κB OR “nuclear factor κB” OR “nuclear factor kappa B”), #3: #1 AND #2; retrieved records: 326). No additional filters and limits were used.

### 2.3. Selection Process

The selection process was conducted in accordance with the Preferred Reporting Items for Systematic Reviews and Meta-analysis (PRISMA) 2000 guidelines [59] and has been registered in the public register of systematic reviews (PROSPERO; ID CRD420261294578). All records were compiled in an Excel file. Duplicate entries were removed manually by two independent reviewers (T.J. and B.R.). The results were compared, and any disagreements were resolved by a third party (M.D.).

Records were selected in an unblinded manner through three stages: title screening, abstract screening, and full text screening. During the title screening stage, titles referring to conditions other than peritoneal fibrosis and peritoneal adhesions were excluded. If eligibility could not be determined based on the title alone, the record was advanced to the next stage. Records that passed title screening underwent abstract screening. In this stage, abstracts unrelated to peritoneal fibrosis or peritoneal adhesions were excluded. Records in which NF-κB was not mentioned in the abstract were also excluded. If item was not specified, the record proceeded to the next step. If eligibility could not be clearly established from the abstract, the record was advanced to the next stage. Records that passed abstract screening underwent full text retrieval. If the full text was not available, the record was excluded. Full text screening was performed manually by two independent reviewers (T.J. and B.R.). At each stage of the selection process, the reviewers’ results were compared, and any disagreements were resolved by a third party (M.D.). No automation tools were used in the study selection process.

### 2.4. Data Collection and Grouping Process

Data collection process was conducted by two reviewers (T.J., who manually extracted data from each record; and M.D., who manually reviewed data extraction). No automation tools were used. The data extraction sheet was designed based on the Cochrane Consumers and Communication Review Group template [60]. The extracted information included: the year of publication, the aim of study, type of study, item of study (peritoneal fibrosis and NF-κB, peritoneal adhesions and NF-κB), and model used (in vitro model, in vivo model). If the study does not represent the experimental or observational study type, the record was excluded. If at least one of the listed items was not investigated in the full text, the record was also excluded. Next, the extracted information was divided into sections for peritoneal fibrosis and peritoneal adhesions. Each section included: tissue and cell model details (type and cell stimulation), animal model details (type and fibrosis/adhesion induction), treatment, main molecular markers, main molecular methods, and outcome description. At the end, funding source and ethical approval data were extracted. The data extraction sheet is available in Supplementary Table S1.

Research articles were grouped based on the main study item as studies on peritoneal fibrosis and studies on peritoneal adhesions. Both fibrosis and adhesion studies were then subsequently grouped into studies on NF-κB-mediated regulation and studies on NF-κB-targeted therapy. Data grouping process was conducted by two reviewers (T.J., who manually grouped records; M.D., who manually reviewed grouping). No automation tools were used. No additional methods were employed to prepare the data for presentation.

### 2.5. Risk of Bias Assessment

Given that most included records represented animal studies, the risk of bias was assessed using the RoB tool for animal intervention studies (SYRCLE’s RoB tool) [61]. The risk of bias assessment was conducted independently by two reviewers (T.J. and N.K.), and any disagreements were resolved by a third party (M.D.). No automation tools were used in this process. The level of risk of bias was considered a source of heterogeneity. Due to substantial heterogeneity among studies, a meta-analysis was not feasible. Consequently, data analysis was descriptive, and the results were summarized in tables.

## 3. Results

### 3.1. Study Selection

**Figure 1:**
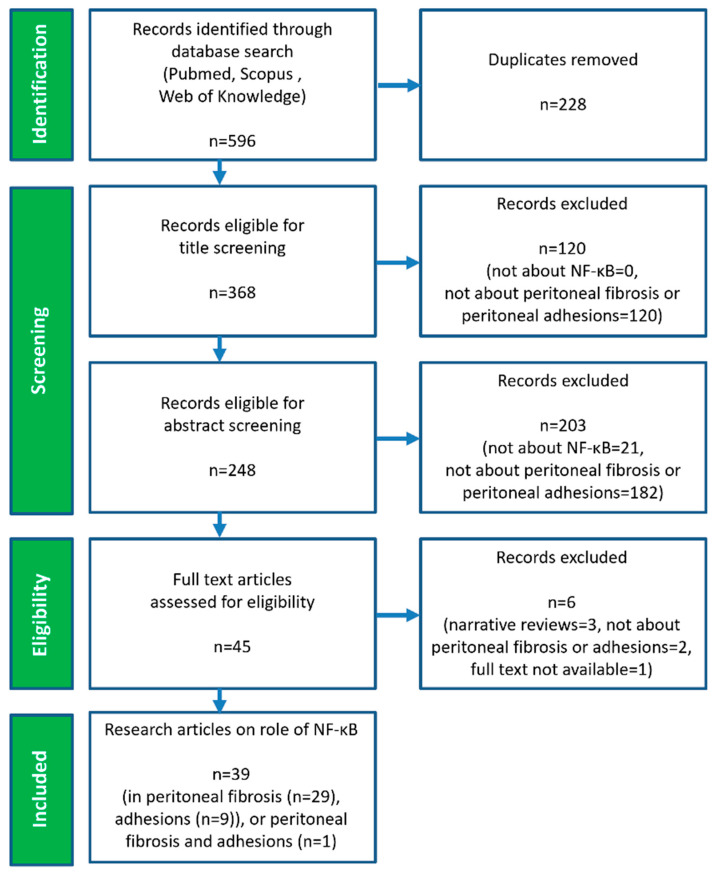
PRISMA flow diagram of records included and excluded from the review.

A total of 596 records were retrieved through the search process and subsequently screened through the selection process, as depicted in Figure 1.

The primary reasons for study exclusion were failure to meet the inclusion criteria, including content unrelated to peritoneal fibrosis or adhesions (a total of 304 records: 120 excluded during title screening, 182 during abstract screening, and 2 during full text screening) or unrelated to NF-κB (21 records excluded during abstract screening). Of the 45 records that proceeded to full text evaluation, two were excluded due to this item discrepancy [62,63], three were excluded because of study type (narrative reviews) [64,65,66], and one was excluded due to lack of full text availability [67]. Records excluded during full text screening are available in Supplementary Table S2. Consequently, 39 records met the inclusion criteria, as research articles addressing the role of NF-κB in peritoneal fibrosis and adhesions, and were included in the final evaluation. These records comprised 29 studies on peritoneal fibrosis, 9 studies on peritoneal adhesions, and only one study addressing both items [10]. This latter study was therefore considered in both groups and discussed in two subsections.

### 3.2. Research Articles on the Role of NF-κB in Peritoneal Fibrosis

#### 3.2.1. NF-κB-Mediated Regulation of Peritoneal Fibrosis

Of the 30 studies on peritoneal fibrosis, 15 studies primarily concerned NF-κB-mediated regulation. These studies are summarized in Table 1.

It can be noted that in 2001, the human peritoneal mesothelial cells were shown to be capable of secreting MCP-1 and IL-8 in response to TNF-α/IL-1β stimulation, as well as in response to the presence of macrophages harvested from effluent drained from patients undergoing PD [68]. The authors proposed that this upregulation may occur via NF-κB signaling; however, no specific pathway was identified [68]. Later studies confirmed that MCP-1 secretion by peritoneal mesothelial cells is suppressed by NF-κB inhibition and also demonstrated the secretion of IL-6 and hyaluronan synthases (HASs) in response to effluent peritoneal dialysate [69].

Also in 2001, it was reported that high glucose concentration, but not mannitol, upregulates MCP-1 secretion by peritoneal mesothelial cell via activation of the protein tyrosine kinase (PTK)/activator protein-1 (AP-1) pathway, but not the protein kinase C (PKC)/NF-κB pathway; and that secreted MCP-1 enhances monocyte migration in mesothelial cell culture [70]. However, a later study showed that high osmolality, induced by addition of either glucose or mannitol, upregulates MCP-1 secretion by activating the nuclear factor of activated T cells 5 (NFAT5)/NF-κB pathway, in which NFAT5 acts as the osmosensitive transcription factor in peritoneal mesothelial cell [71]. Further study demonstrated that the NFAT5/NF-κB pathway is activated not only in stimulated peritoneal mesothelial cell culture but also in mesothelial cell collected by biopsy from uremic patients undergoing PD. This activation leads to increased MCP-1 secretion and enhanced macrophage migration to the peritoneum [40]. Additionally, a study in peritoneal mesothelial cell cultures showed that glucose, but not mannitol, induces upregulation of inflammatory (MCP-1) marker, fibrosis mediator (TGF-β1), and fibrosis markers (α-SMA, fibronectin) via activation of NF-κB signaling through the Toll-like receptors (TLR) myeloid differentiation primary response 88 (MyD88)-dependent cascade, specifically through TLR 4 [72]. High glucose concentration also upregulates fibrosis markers (fibronectin, collagen I, and plasminogen activation inhibitor-1 (PAI-1)) in rat peritoneal mesothelial cell cultures [73] as well as upregulates inflammatory markers (MCP-1, IL-6, TNF-α) and fibrosis mediator (TGF-β1) in rat peritoneal mesothelial cell culture and uremic rats, respectively [74]. Glucose additionally downregulates peroxisome proliferator-activated receptor γ (PPARγ) expression [73] and upregulates prostaglandin E2 (PGE_2_) receptor subtype 4 expression [75]. Activation of PPARγ downregulates glucose-stimulated fibrosis by inhibiting AP-1 and NF-κB activity, although the direct signaling pathway has not been fully elucidated [73]. Similarly, activation of peroxisome proliferator-activated receptor β/δ (PPARβ/δ) downregulates glucose-stimulated inflammation by inhibiting transforming growth factor-β-activated kinase 1 (TAK1) and NF-κB activity, thereby suppressing the TAK1/NF-κB signaling pathway [74].

In TGF-β1-induced in vitro models, TAK1 inhibition in human peritoneal mesothelial cell culture—both commercially available and effluent-derived—has been reported to downregulate MMT markers (loss of E-cadherin, vimentin) and fibrosis markers (fibronectin, PAI-1), by reducing the transcriptional activity of multiple factors, including Smads, AP-1, Snail, and NF-κB. Consequently, the TAK1/NF-κB signaling pathway has been proposed to contribute to MMT in response to TGF-β1/IL-1β stimulation [76]. Furthermore, Src inhibition in human peritoneal mesothelial cell cultures and in a non-uremic rat model was shown to downregulate fibrosis markers (α-SMA, fibronectin, collagen I) through inhibition of the TGF-β/Smad pathway. In this model, Src inhibition also downregulated inflammation markers (MCP-1, IL-1β, IL-6, TNF-α) and fibrosis mediator (TGF-β1) by inhibiting NF-κB activation [77]. In similar in vitro and in vivo models, TGF-β1 was shown to stimulate autophagic activity, which contributed to MMT (loss of E-cadherin) and fibrosis (fibronectin, collagen I) marker upregulation via the TGF-β1/Smad pathway, as well as inflammatory marker (MCP-1, IL-1β, IL-6) upregulation via the crosstalk between signal transducer and activator of transcription 3 (STAT3) and NF-κB pathways [78]. Additionally, in a TGF-β1-induced in vitro model combined with a non-uremic mouse model, genetic deletion or pharmacological inhibition of the histone methyltransferase enhancer of zeste homolog 2 (EZH2) suppressed TGF-β1-induced upregulation of MMT (α-SMA, but not E-cadherin) and fibrosis (collagen I) markers by inhibiting the TGF-β/Smad pathway. Given that elevated EZH2 level was detected in the peritoneum and effluent from patients undergoing PD, its inhibition also ameliorated peritoneal inflammation and fibrosis by downregulation of inflammation markers (MCP-1, IL-1β, IL-6, TNF-α), fibrosis mediator (TGF-β1), and angiogenesis marker (VEGF) via suppressing STAT3 and NF-κB activation [79]. Another study in a non-uremic rat model without TGF-β1 stimulation demonstrated that STAT3 and NF-κB activation were similarly suppressed by knockdown of the epidermal growth factor (EGF) receptor (EGFR), resulting in downregulation of fibrosis (collagen I) and inflammatory (MCP-1) markers [80].

Similarly to EZH2, expression of the PGE2 receptor subtype 4 [75] and the stimulator of interferon genes (STING) [10] was increased in human peritoneal biopsies from patients undergoing PD. Inhibition of the this PGE2 receptor downregulated MMT (vimentin) and fibrosis (collagen I, fibronectin) markers by inhibiting activation of the NLR family pyrin domain containing 3 (NLRP3) inflammasome and also downregulated inflammatory markers (MCP-1, IL-1β, TNF-α) by suppressing NF-κB activation [75]. In contrast, genetic deletion or inhibition of STING predominantly downregulated inflammatory markers (MCP-1, IL-1β, IL-6, TNF-α) by inhibiting NF-κB activation during sterile and infection-induced peritoneal injury. STING inhibition also attenuated macrophage recruitment to the peritoneum, reduced macrophage-driven MMT and fibrogenesis by downregulating fibrosis mediator (TGF-β1), MMT marker (α-SMA), and fibrosis marker (fibronectin). STING inhibition also downregulated angiogenesis markers (VEGF, chemokine (C-X-C motif) ligand 1 (CXCL1)) via an independent mechanism [10].

#### 3.2.2. NF-κB-Targeted Therapy for the Prevention of Peritoneal Fibrosis

Of the 30 studies on peritoneal fibrosis, 15 studies were also primarily concerned with NF-κB-targeted therapy. These studies are summarized in Table 2.

It can be observed that in 2003, simvastatin was demonstrated to stimulate fibrinolytic capacity (tissue-type plasminogen activator (t-PA)) and suppress the procoagulant activity (PAI-1) of human peritoneal mesothelial cells in an in vitro model stimulated by TNF-α. The authors proposed inhibition of AP-1 and NF-κB activity as a molecular explanation for this antifibrotic effect [81]. However, this was a single NF-κB-targeted therapy study using the TNF-α-stimulated model.

In high-glucose-induced in vitro models of peritoneal fibrosis, prednisolone was shown to suppresses osmotic stress-induced MCP-1 secretion by inhibiting the PKC/NF-κB pathway [82], while astaxanthin was shown to prevent glucose-induced MMT (loss of E-cadherin), fibrosis (α-SMA), and inflammatory (TNF-α) marker upregulation as well as fibrosis mediator (TGF-β) upregulation by inhibiting the ROS/NF-κB pathway [83]. In both glucose-induced in vitro and non-uremic in vivo mouse models, parthenolide suppressed MMT (loss of E-cadherin), fibrosis (fibronectin, collagen I, α-SMA), and inflammatory (MCP-1, IL-6, TNF-α) markers as well as fibrosis mediator (TGF-β) upregulation through inhibiting the TGF-β1/Smad pathway and NF-κB activation, suggesting a TGF-β/NF-κB crosstalk and common NF-κB/TGF-β/Smad signaling axis [41]. In comparable models using rats rather than mice, polydatin was shown to mitigate glucose-induced MMT (loss of E-cadherin), fibrosis (collagen I, α-SMA), angiogenesis (VEGF), and inflammatory (IL-1β, interleukin-18 (IL-18)) marker upregulation, as well as fibrosis mediator (TGF-β) and ROS production by inhibiting the NLRP3/NF-κB pathway [84]. Similarly, in a glucose degradation product-induced non-uremic in vivo mouse model, the active compound—in this case, (−)-epigallocatechin-3-gallate (EGCG)—reduced upregulation of angiogenesis marker (VEGF), fibrosis mediator (TGF-β), and inflammatory markers (MCP-1), as well as ROS production, by inhibiting NF-κB activity [85]. Moreover, in both glucose-induced in vitro and uremic in vivo mouse models, Shenbing Decoction III (SBD III) and its key active component—apigenin—were shown to reduce MMT marker (loss of E-cadherin), fibrosis markers (fibronectin, collagen I, α-SMA), and fibrosis mediator (TGF-β) upregulation by inhibiting the TAK1/p38MAPK/NF-κB pathway. In this study, only p38 mitogen-activated protein kinase (p38) was considered within the mitogen-activated protein kinase (MAPK) family [86].

In a TGF-β1-induced in vitro model of peritoneal fibrosis, arctigenin was shown to suppress MMT marker (loss of E-cadherin) and fibrosis markers (fibronectin, collagen I, α-SMA, PAI-1), by inhibiting IκBα phosphorylation and activating the adenosine monophosphate-activated protein kinase (AMPK)/NF-κB pathway [87]. While in an interferon-γ (IFN-γ)-induced in vitro model, pemafibrate inhibited TGF-β1 production and consequently reduced fibrosis (fibronectin) and inflammatory (IL-1β, IL-6, TNF-α) marker upregulation by inhibiting AP-1 and NF-κB activity, particularly through stabilization of IκBα. However, the in vivo model used in this study did not investigated the role of NF-κB [88].

In a chlorhexidine gluconate-induced, non-uremic in vivo mouse model of peritoneal fibrosis, calcitriol was shown to reduce upregulation of fibrosis markers (collagen III, α-SMA), inflammatory marker (MCP-1), and fibrosis mediator (TGF-β), as well as macrophage infiltration, by inhibiting the TGF-β1/Smad pathway and NF-κB activation [89]. In a similar model using rats, suramin was also demonstrated to reduce fibrosis (fibronectin, collagen I, α-SMA), inflammatory (MCP-1, IL-1β, IL-6, TNF-α), and angiogenesis (VEGF) markers upregulation, as well as fibrosis mediator (TGF-β) upregulation and macrophage infiltration, through the same pathways [90]. Likewise, in a comparable mouse model, chondroitin sulfate suppressed inflammatory markers (MCP-1, IL-1β) upregulation and macrophage infiltration by inhibiting NF-κB activation and likely the TGF-β/Smad pathway, given that this study assessed phosphorylated Smad2/3 but not TGF-β levels directly [91].

In a more comprehensive experimental design incorporating non-uremic and uremic models—both in vitro and in vivo—as well as an lipopolysaccharide (LPS)-induced in vitro model and a chlorhexidine gluconate-induced in vivo rat model, the role of dulaglutide in preventing peritoneal fibrosis in the context of CDK was examined in detail. Dulaglutide was demonstrated to reduce fibrosis marker (fibronectin, collagen I, α-SMA) upregulation by inhibiting the TGF-β/Smad pathway and to attenuate oxidative stress by inhibiting signaling involving dipeptidyl peptidase 4 (DPP4), glucagon-like peptide 1 receptor (GLP-1R), and nuclear factor erythroid 2-related factor 2 (Nrf2). In this study, dulaglutide also reduced inflammatory marker (TNF-α) upregulation not only through inhibition the DPP4/GLP-1R/NF-κB pathway, but also via suppression of the TGF-β/NF-κB crosstalk [92]. In addition, in an LPS-induced in vitro model of peritoneal fibrosis, melatonin was shown to suppress MMT (loss of E-cadherin, vimentin) and fibrosis (α-SMA) markers upregulation by inhibiting the TLR4/AP-1 and TLR4/NF-κB/Snail pathways, where Snail acts as a repressor of E-cadherin expression [93]. Similarly, dioscin was demonstrated to attenuate MMT (loss of E-cadherin, vimentin), fibrosis (fibronectin, collagen I, α-SMA), and inflammatory (IL-1β, IL-6, TNF-α) marker upregulation by inhibiting the TGF-β1/Smad and TLR4/MyD88/NF-κB pathways [94].

Based on findings related to NF-κB-mediated regulation, PPARβ/δ [74] and STING [10] have also been proposed as promising therapeutic targets for preventing PD-associated peritoneal deterioration; however, due to the pioneering nature of these studies [10,74], they were incorporated into the NF-κB-mediated regulation subsection.

### 3.3. Research Articles on the Role of NF-κB in Peritoneal Adhesions

#### 3.3.1. NF-κB-Mediated Regulation of Peritoneal Adhesions

Of the 10 studies on peritoneal adhesions, four primarily examined NF-κB-mediated regulation of peritoneal adhesion formation. These studies are summarized in Table 3.

It can be noted that in 2009, normal and adhesion-derived peritoneal mesothelial cells were shown to be capable of increasing inducible nitric oxide synthase (iNOS) expression through a hypoxia-induced mechanism involving NF-κB activation [95].

The role of chemokine (C-C motif) receptor 8 (CCR8) in the development of peritoneal adhesions was investigated in an in vitro mouse macrophage culture model and three in vivo mouse models (cecal cauterization, cecal abrasion, and ischemic buttons models). In vitro, CCR8 gene deletion or pharmacologic inhibition reduced LPS-induced upregulation of inflammatory markers (IL-6, IL-10, TNF-α) and macrophage migration (chemokine (C-C motif) ligands 1 and 8 (CCL1, CCL8)). In vivo, CCR8 deficiency decreased adhesion formation, as measured by adhesion score. The authors proposed that this effect is mediated through inhibition of the TLR4/NF-κB and TLR4/MAPK/AP-1 pathways, where MAPK family included extracellular signal-regulated kinase (ERK), c-Jun N-terminal kinase (JNK), and p38 [96]. Subsequent study using an in vivo rat model of cecal cauterization further specified that peritoneal adhesion formation is driven by upregulation of inflammatory markers (IL-6, TNF-α, CXCL1, chemokine (C-X-C motif) ligand 1 (CXCL2)) through activation of the TLR4/MyD88/NF-κB pathway [97].

The role of STING in peritoneal adhesion formation was evaluated in an in vivo mouse model induced by surgically generated ischemic buttons. In the ischemic button tissues, increased level of STING, TANK-binding kinase 1 (TBK1), interferon regulatory factor 3 (IRF3), and phosphorylated IκBα was observed in STING-intact mice. In contrast, STING-deficient mice showed reduced adhesion formation (adhesion score), along with decreased level of inflammatory markers (MCP-1 also referred to as chemokine (C-C motif) ligand 2 (CCL2), chemokine (C-C motif) ligand 2 (CCL5)) and interferon-stimulated proteins (interferon gamma-induced protein 10 (IP-10), interferon-induced protein with tetratricopeptide repeats 1 (IFIT1), ubiquitin-specific protease 18 (USP18), Mx2). These findings indicate that STING deficiency attenuates peritoneal adhesion formation by inhibiting the TBK1/IRF3 pathway and NF-κB activation [10].

#### 3.3.2. NF-κB-Targeted Therapy for the Prevention of Peritoneal Adhesions

Of the 10 studies on peritoneal adhesions, 6 examined NF-κB-targeted therapy for the prevention of peritoneal adhesion formation. These studies are summarized in Table 4.

It can be noted that in 2015, cholecalciferol was shown to reduce injury-induced inflammation (inflammation score) and adhesion formation (adhesion score) by inhibiting NF-κB activity in an in vivo rat model of uterine cauterization [98]. Subsequently, the effect of gallic acid was investigated using an in vivo rat model of cecal and parietal peritoneum abrasion. In this model, gallic acid was shown to reduce injury-induced adhesion formation (adhesion score) and upregulation of inflammatory markers (IL-6, TNF-α) and fibrosis mediator (TGF-β) by inhibiting NF-κB activation [99]. In a similar in vivo rat model, androstenediol was demonstrated to reduce upregulation of inflammatory marker (high mobility group box 1 (HMGB1)), fibrosis marker (α-SMA), fibrosis mediator (TGF-β), and thus adhesion formation (adhesion score). It also attenuated oxidative stress by decreasing malondialdehyde (MAD) level and increasing superoxide dismutase (SOD) activity. The authors proposed that these effects are mediated through inhibiting the TLR4/NF-κB pathway [100]. In the same year, sodium butyrate was shown to reduce injury-induced upregulation of inflammatory markers (MCP-1, TNF-α), angiogenesis markers (vascular score, VEGF, CXCL1), and fibrosis mediator (TGF-β) by inhibiting NF-κB activity in an in vivo intra-abdominal foreign body mouse model [101].

The most recent bioengineering advances in biocompatible anti-adhesion scaffolds [102] and biomimetic enzymes with antioxidant and anti-inflammatory properties [103] were published in 2024 and 2025, respectively. In an in vivo rat model of peritoneal injury induced by point hemorrhage, an anti-adhesion membrane composed of poly(lactic-co-glycolic acid) (PLGA) significantly reduced injury-induced upregulation of fibrosis markers (collagen I, collagen III, α-SMA) and adhesion formation (adhesion score) by activating Nrf2 phosphorylation and inhibiting NF-κB activation. In this study, the effects of the PLGA-based membrane on tendon adhesion formation were also evaluated, and the membrane was characterized in detail in vitro; however, these in vitro experiments did not assess peritoneal adhesion formation [102]. In a combined in vitro and in vivo approach to preventing peritoneal adhesions by using biomimetic hydrogel microsphere-encapsulated SOD enzyme (L-CMH/CD) was reported. In vitro, cultured mouse peritoneal fibroblasts exhibited reduced LPS-induced responses while, in vivo, a mouse model of cecal and parietal peritoneal abrasion showed attenuation of injury-induced changes. These effects included decreased upregulation of inflammatory markers (IL-1β, IL-18, TNF-α), fibrotic marker (α-SMA), oxidative stress (ROS), and adhesion formation (adhesion score), mediated through inhibition of the piezo-type mechanosensitive ion channel component 1 (Piezo1)/NF-κB pathway [103].

### 3.4. Risks of Bias Assessment

**Figure 2:**
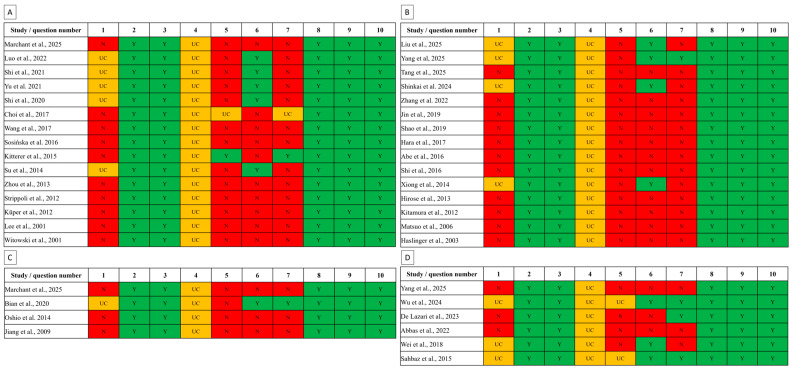
Bias assessment of animal studies according to SYRCLE tool in studies on (**A**,**B**) peritoneal fibrosis and (**C**,**D**) peritoneal adhesions in the context of (**A**,**C**) NF-κB-mediated regulation and (**B**,**D**) NF-κB-targeted therapy. Question numbers correspond with SYRCLE tool [10,40,41,68,69,70,71,72,73,74,75,76,77,78,79,80,81,82,83,84,85,86,87,88,89,90,91,92,93,94,95,96,97,98,99,100,101,102,103]. Scores: Y: yes (marked in green); N: no (marked in red); UC: unclear (marked in orange).

The risks of bias in individual articles are depicted in plots presented in Figure 2.

All included studies, regardless of whether they addressed peritoneal fibrosis or peritoneal adhesions, as well as NF-κB–mediated regulation or NF-κB–targeted therapy, showed no risk of bias in domains of group similarity (question 1), adequate allocation in groups (question 3), adequate outcome assessment (question 8), free of selective outcome reporting (question 9), and free of other problems (question 10). However, all included studies demonstrate some risk of bias due to random housing (question 4), as housing descriptions in these study was incomplete and thus risks were assessed as ‘uncreal’. Each study demonstrates fewer or greater problems with risks due to adequate sequencing (question 1), resulting in 13 studies assessed as ‘unclear’ and 27 as ‘no’ in this domain. Similarly, almost every study reported problems with blinded intervention (question 5), so 36 studies were assessed as ‘no’, 2 as ‘unclear’, and only 1 as ‘yes’. Higher risk heterogeneity was observed for random selection of animals (question 6) and blinded outcome assessment (question 7), with some divergence. Among studies on NF-κB-mediated regulation in peritoneal fibrosis, 5 were assessed as ‘yes’ and 10 as ‘no’ when answering question 6. In this group, 1 study was assessed as ‘yes’, 1 as ‘unclear’, and 13 as ‘no’ when answering question 7. Among studies on NF-κB-targeted therapy in peritoneal fibrosis, 4 were assessed as ‘yes’ and 11 as ‘no’ when answering question 6. In this group, 1 study was assessed as ‘yes’, and 14 as ‘no’ when answering question 7. When considering peritoneal adhesions in the context of NF-κB-mediated regulation, one study was assessed as ‘yes’ and three as ‘no’ when answering both questions 6 and 7. When considering peritoneal adhesions in the context of NF-κB-targeted therapy, three studies were assessed as ‘yes’ and three as ‘no’ when answering question 6 as well as three studies were assessed as ‘yes’, and three as ‘no’ when answering question 7; however, it was not the same studies.

Substantial heterogeneity arises from the diversity of the research questions addressed and the experimental models employed. The models used in human and animal studies on peritoneal fibrosis and peritoneal adhesions are summarized in Table 5 and Table 6, respectively. Across all included studies, no two investigated an identical research question using the same experimental model, a finding that is particularly evident among studies evaluating NF-κB-targeted therapies in both peritoneal fibrosis and peritoneal adhesion research. Moreover, none of the included studies—particularly those investigating NF-κB-targeted therapies—represent human clinical trials. This suggests that although regulation of NF-κB-mediated inflammatory responses appears to be a promising strategy for the prevention and treatment of peritoneal fibrosis or peritoneal adhesion formation, its clinical application remains distant.

## 4. Discussion

**Figure 3:**
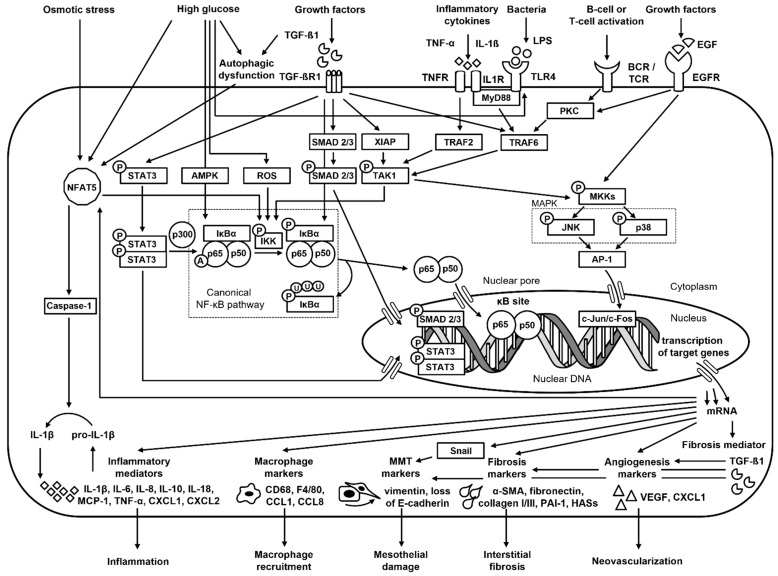
A simplified schema of the NF-κB signaling pathway in the peritoneal mesothelium affected by fibrosis and adhesion formation. Footnotes: α-SMA—α-smooth muscle actin; a—acetyled; AMPK—adenosine monophosphate-activated protein kinase; AP-1—activator protein 1; BCR—B-cell receptor; CD68—cluster of differentiation 68 expressed by cells in the monocyte/macrophage lineage; CCL—chemokine (C-C motif) ligand; CXCL—chemokine (C-X-C motif) ligand; EGF—epidermal growth factor; EGFR—epidermal growth factor receptor; F4/80—surface protein of mouse macrophage; HASs—hyaluronan synthases; IκBα—inhibitor kappa B α; IL—interleukin; IL1R—interleukin-1 receptor; IKK—IκB kinase; JNK—c-Jun N-terminal kinase; LPS—lipopolysaccharide; MAPK—mitogen-activated protein kinase; MCP-1—monocyte chemoattractant protein 1; MAKK—mitogen-activated protein kinase kinase; MMT—mesothelial-to-mesenchymal transition; MyD88—myeloid differentiation primary response 88; NF-κB—nuclear factor kappa B (subunit p50; subunit p65 (Rel A)); NFAT5—nuclear factor of activated T cells 5; p-—phosphorylated; p38—p38 mitogen-activated protein kinase; PAI-1—plasminogen activator inhibitor type 1; PKC—protein kinase C; pro-—inactive precursor; ROS—reactive oxidative species; STAT3—signal transducer and activator of transcription 3; TAK1—TGF-β-activated kinase 1; TCR—T-cell receptor; TGF-β1—transforming growth factor-β1; TGF-βR1—transforming growth factor β receptor 1; TLR4—Toll-like receptor 4; TNF-α—tumor necrosis factor-α; TNFR—tumor necrosis factor receptor; u-—ubiquitinated; VEGF—vascular endothelial growth factor.

Previous studies have shown that multiple signaling pathways are involved in the initiation and progression of peritoneal fibrosis, including the TGF-β1 [27,30,32,44], EGF [104], and STAT3 [105] pathways; however, far fewer studies have been devoted to the mechanisms underlying peritoneal adhesions [15,16]. In the peritoneal fibrosis studies included in this systematic review, activation of, predominately, the NFAT5/NF-κB [40,71], PKC/NF-κB [70], TLR4/MyD88/NF-κB [72], and TAK1/p38MAPK/NF-κB [86] pathways, as well as TGF-β/NF-κB [41,92] and STAT3/NF-κB [78] crosstalk, were demonstrated. In contrast, in the context of peritoneal adhesions, predominately bacteria-induced TLR4/NF-κB [96,100], and more specifically TLR4/MyD88/NF-κB [97], were investigated. These pathways are summarized in Figure 3.

It can be observed that, among these pathways, NFAT5 acts as an osmosensitive transcription factor [71]. Activation of NFAT5 by high osmolarity regulates gene expression, among other mechanisms, by promoting IκBα degradation and p65 nuclear translocation [106]. Crosstalk between the NFAT5/NF-κB, TGF-β/Smad, and STAT3 pathways may be suggested through the regulation of autophagy, given that autophagic dysfunction stimulate NFAT5 [106] and that blockade of autophagy prevents peritoneal fibrosis by suppressing activation of the NF-κB, STAT3, and TGF-β1/Smad pathways [78]. Interestingly, in several studies, activation of NF-κB was described together with activation of the TGF-β/Smad pathway [77,78,79,89,90,91,94], and direct TGF-β/NF-κB crosstalk was also proposed [41,92]. Moreover, crosstalk between the STAT3 and NF-κB pathways has been suggested [78], as STAT3 may sustain NF-κB activation via p300-mediated acetylation [107]. Similar crosstalk between EGF and NF-κB may be expected, as the AP-1 complex and NF-κB were concurrently activated in multiple studies on both peritoneal fibrosis [73,76,81,88,93] and peritoneal adhesion [96]. In studies reporting simultaneous activation of AP-1 and NF-κB, experimental systems included rat peritoneal mesothelial cells stimulated with glucose [73], mouse peritoneal macrophages stimulated with LPS [96], and human peritoneal mesothelial cells stimulated with TGF-β1 [76,77], IL-1β [76], TNF-α [81], IFN-γ [88], or LPS [93], as well as non-CKD rat models treated with chlorhexidine gluconate [77]. The EGF/NF-κB interaction may be mediated through the PKC/NF-κB [70], TLR4/MyD88/NF-κB [72], and TAK1/p38MAPK/NF-κB [86] pathways. In the proposed PKC/NF-κB pathway [70], stimulation of the EGFR may activate PKC/TRAF6/TAK1 signaling, in which TAK1 phosphorylates IKK, leading to NF-κB activation [108]. In the TLR4/MyD88/NF-κB pathway [72], EGF and NF-κB pathways may crosstalk at the level of TAK1 [108]. In contrast, in the proposed TAK1/p38MAPK/NF-κB pathway [86], the involvement of p38MAPK in NF-κB activation remains unclear, and this pathway may be more accurately described as crosstalk between the TAK1/p38 MAPK/AP-1 and TAK1/IKK/NF-κB pathways. Moreover, EGFR knockdown attenuates peritoneal fibrosis by suppressing STAT3 and NF-κB activation [80], suggesting not only EGF/NF-κB but also EGF/STAT3 crosstalk. It can be observed that similar signaling pathways, particularly the TLR4/NF-κB [96,97] pathway, may play a role in peritoneal adhesion formation. However, the marked imbalance in both the number and depth of studies on peritoneal fibrosis versus peritoneal adhesions indicates that the role of NF-κB in adhesion formation remains under investigated.

Particular attention should be paid to TGF-β/NF-κB crosstalk [41,92], where both NF-κB can be activated by TGF-β and NF-κB can mediate transcription activation of TGF-β target genes [109]. Given that in peritoneal membrane, TGF-β1 induced MMT [21,22], fibroblast differentiation into myofibroblasts [27], fibroblast activation [27], and vascular endothelial cells activation [27,30], these processes are pivotal in the progression of peritoneal fibrosis. Although both MMT and interstitial fibrosis can be mediated through TGF-β1-dependent, NF-κB-independent pathways [27,30,32,44] and through NF-κB-dependent, TGF-β1-independent pathways [38,39,40] in mesothelial cells, it should be noted that crosstalk between the TGF-β and NF-κB has recently been reported in other cell types [109,110,111,112,113,114]. However, the specific mechanism linking NF-κB and TGF-β remain incompletely understood, and published results are inconsistent [109]. For example, in interstitial polymorphonuclear neutrophils [110] chondrosarcoma cells [111], and pancreatic duct cells [112], NF-κB can be activated by TGF-β and mediate transcriptional activation of TGF-β target genes. In contrast, in intestinal lamina propria mononuclear cells, NF-κB can be repressed by TGF-β through a negative feedback loop [113]. In squamous cell carcinoma cells, NF-κB can inhibit TGF-β/Smad signaling by inducing Smad7 expression [114]. Importantly, in human peritoneal mesothelial cells exposed to high glucose, NF-κB can directly bind to the TGF-β1 promoter to transcriptionally regulate TGF-β1 expression [41]. In contrast, in human pleural mesothelial cells treated with uremic toxin and LPS, TGF-β1 upregulated NF-κB activity [92]. Moreover, in human peritoneal mesothelial cells treated with TGF-β1, it upregulated IκBa phosphorylation and thus NF-κB activity, indicating activation of the NF-κB pathway by TGF-β1 [87]. Questions also remain regarding TGF-β/NF-κB crosstalk in studies in which NF-κB activation was reported alongside activation of the TGF-β/Smad pathway [77,78,79,89,90,91,94], especially since NF-κB activation by TGF-β can be mediated in both Smad-dependent and Smad-independent manner [109]. Moreover, activation of the ROS/TGF-β/Smad pathway leads to excessive release of macrophage extracellular traps, which are in turn associated with enhanced MMT, angiogenesis, and inflammation in the mouse peritoneal mesothelium exposed to high-glucose PD fluid [115]. Similarly, in animal models stimulated with high-glucose PD fluid—both mouse [84] and rat [85]—activation of the ROS/NF-κB pathway led to increased production of MMT [84], fibrosis [84], angiogenesis [84], and inflammatory markers [84,85]. These in vivo observations are consistent with an in vitro study using rat peritoneal mesothelial cell cultures stimulated with high glucose, which showed that activation of the ROS/NF-κB pathway led to increased expression of MMT, fibrosis, and inflammatory markers [83]. Taken together, these findings suggest that ROS, through its interaction with both TGF-β and NF-κB, may represent an important component of TGF-β/NF-κB crosstalk and warrants further investigation in the context of peritoneal fibrosis. Although most reports indicate that TGF-β activates NF-κB, TGF-β1 may also repress NF-κB signaling [109]. In studies reporting simultaneous activation of TGF-β/Smad and NF-κB, experimental models included human peritoneal mesothelial cells stimulated with TGF-β1 [77,78,79] or LPS [94], non-CKD rat models [77,78,90] and mouse models [79,89,91] treated with chlorhexidine gluconate, as well as mouse models exposed to glucose-based peritoneal dialysis solution [79]. Together, these findings suggest that TGF-β/NF-κB crosstalk varies depending on the type of stimulation, and therefore on the conditions under which these pathways operate.

NF-κB is activated by cellular exposure to various stimuli, including high glucose, LPS, inflammatory cytokines (IL-1, TNF-α), growth factors, viral infection or viral gene products, ultraviolet radiation, and B-cell or T-cell activation [43,48,116]. In the reviewed studies, NF-κB activation was demonstrated in vitro in human [40,41,71,72,84] and rat [41,73,74,75,82,83] peritoneal mesothelial cells exposed to glucose [40,41,71,72,73,74,75,82,83,84] and osmotic stress [71,82], as well as in human peritoneal mesothelial cells exposed to effluent from glucose-based PD [40,68,69] and to glucose-based PD solution [86]. Similarly, in vivo studies demonstrated NF-κB activation in mouse mesothelial cells exposed to glucose-based PD solutions [41,79,85,86] and glucose degradation products such as methylglyoxal (MGO) [85], as well as in rat mesothelial cells exposed to glucose-based PD solutions [74,75,77,80,84]. Moreover, NF-κB was activated in human peritoneal mesothelial cells directly exposed to LPS [10,93,94], inflammatory cytokines (TNF-α [68], IL-1β [68,76], IFN-γ [88]), growth factor (TGF-β1 [76,77,79,81,87]), and activated macrophage-conditioned media (AMCM), which is rich in inflammatory cytokines and growth factors [10]. Correspondingly, in vivo studies showed NF-κB activation in mouse mesothelial cells exposed to bacteria [10] and in rat mesothelial cells exposed to LPS [74]. NF-κB activation in vivo was also induced by peritoneal irritation. In peritoneal fibrosis studies, irritation was produced by exposure to chlorhexidine gluconate in mice [10,79,89,91] and rats [90,92]. In peritoneal adhesion studies, more severe mechanical stress was induced by intra-abdominal surgical interventions in mice [10,96,101,103] and rats [97,98,99,100,102]. Depending on the model, this mechanical stress either involved exposure to gastrointestinal bacteria and LPS—such as in cecal abrasion [96,99,100,103] or cecal cauterization [96,97] models—or was applied under sterile conditions, including ischemic buttons [10,96], uterine cauterization [98] foreign body implantation [101], and peritoneum puncture with point hemorrhage [102]. Finally, NF-κB activation in animal models of peritoneal fibrosis should be interpreted differently depending on whether non-CKD models [10,41,75,77,80,84,85,86,89,90,91] or CKD models—such as those induced by 5/6 nephrectomy [10,74] or administration of uremic toxin (e.g., p-Cresol) [92]—were used. CKD models more closely mimic the clinical setting of patients undergoing peritoneal dialysis and therefore provide a more relevant platform for translational research [92].

It can be observed that routinely used PD solutions are hyperglycemic, hyperosmotic, and acidic [12,13,14], and that several components of the PD solution—such as glucose and glucose degradation products, which serve as osmotic agents—activate NF-κB in the peritoneal mesothelium. This activation results in upregulation of inflammatory mediators (MCP-1 [40,41,68,69,71,72,74,75,78,79,80,82,85], IL-1β [75,78,79,84], IL-6 [41,69,74,78,79], IL-8 [68], IL-18 [84], TNF-α [41,74,75,79,83]), macrophages recruitment (CD68 [40,78]), and fibrosis mediator (TGF-β1 [41,72,74,78,79,83,84,85,86]). This upregulation promotes inflammation and initiates a cascade of cellular and molecular changes leading to peritoneal fibrosis. In this cascade, TGF-β1, the principal mediator of peritoneal fibrosis, binds to TGF-β receptor 1 (TGF-βR1), induces phosphorylation of downstream signaling molecule (Smad2/3 [41], Smad3 [78,79]), and thereby promotes expression of target genes. This leads to upregulation of MMT markers (loss of E-cadherin [41,78,79,83,84,86], vimentin [75]), indicating mesothelial damage; fibrosis markers (α-SMA [41,72,79,83,84,86], fibronectin [41,72,73,75,78,86], collagen I [41,73,75,78,79,80,84,86], PAI-1 [73,76,87], HASs [69]), indicating interstitial fibrosis; and angiogenesis markers (VEGF [79,83,84,85]), reflecting neovascularization. Although most of the reviewed studies demonstrated that exposition of peritoneal mesothelial cells to high glucose level activates NF-κB [40,41,71,72,73,74,75,82,83,84], one study showed that glucose upregulated MCP-1 secretion via the PTK/AP-1 pathway rather than the PKC/NF-κB pathway. The authors suggested that this discrepancy may be explained the transient nature of glucose-induced NF-κB activation, which occurs early after stimulation, whereas NF-κB was assessed after 1, 3, and 5 days in this experiment [70]. Moreover, this study showed that an equivalent concentration of mannitol had no significant effect on MCP-1 secretion, suggesting that glucose-induced MCP-1 upregulation is independent of osmolality [70]. In contrast, the osmotic stress-induced MCP-1 secretion was confirmed to be mediated through activation of the NFAT5/NF-κB pathway [71]. Thus, NF-κB can be activated in peritoneal mesothelium not only by glucose but also by osmotic stress [71], and both glucose- and osmotic-induced MCP-1 secretion can be suppressed by prednisolone [82]. In addition, low pH was shown to inhibit NF-κB activity while simultaneously upregulating MCP-1 secretion in human peritoneal mesothelial cell exposed to acidic PD solution [117]. During PD, direct exposure to acidic solution, high glucose, glucose degradation products, osmotic stress, or their combination leads to persistent peritoneal inflammation [15,74], which in turn drives progressive morphological and functional alterations in the peritoneal membrane.

Interestingly, in a non-CKD mouse model of peritoneal fibrosis induced by glucose-based PD solution and glucose degradation products, mice developed fatal colonic adhesions, and intra-abdominal cocoon-like clusters of connective tissue were observed [85], suggesting a strong response to experimental irritation. Chlorhexidine gluconate-induced peritoneal irritation was shown to activate NF-κB, resulting in upregulation of inflammatory mediators (MCP-1 [77,78,79,89,90,91], IL-1β [77,78,79,90,91], IL-6 [77,78,79,90], TNF-α [77,79,90]), macrophage infiltration (CD68 [78,90], F4/80 [89,91]), fibrosis mediator (TGF-β1 [77,79,89,90]), MMT markers (loss of E-cadherin [78,79]), fibrosis markers (α-SMA [77,79,89,90], fibronectin [77,78,90], collagen I [77,78,79,90], collagen III [89]), and angiogenesis markers (VEGF [79,90]). Similarly, LPS-induced peritoneal irritation activates NF-κB, leading to upregulation of inflammatory mediators (IL-1β [94], IL-6 [94], TNF-α [94]), fibrosis mediator (TGF-β1 [94]), MMT markers (loss of E-cadherin [93,94], vimentin [93,94]), and fibrosis markers (α-SMA [93,94], fibronectin [94], collagen I [94]). When both stimuli were combined, NF-κB-dependent upregulation of inflammatory mediators (MCP-1 [10], IL-1β [10], IL-6 [10], TNF-α [10,92]), fibrosis mediator (TGF-β1 [10,92]), fibrosis markers (α-SMA [10,92], fibronectin [10,92], collagen I [92]), and angiogenesis markers (VEGF [10], CXCL1 [10]) was observed. In addition, exposition to LPS caused marked damage to the peritoneal mesothelial cells and their increased permeability [92]. The stronger inflammatory response likely reflects recruitment of inflammatory cells during bacterial infection, which further amplifies mediator production [81]. In peritoneal adhesion studies, intra-abdominal surgery-induced mechanical stress similarly activated NF-κB, resulting in upregulation of inflammatory mediators (MCP-1 [101], TNF-α [101]), fibrosis mediator (TGF-β1 [101]), fibrosis markers (α-SMA [102], collagen I [102], collagen III [102]), and angiogenesis markers (VEGF [101], CXCL1 [101]). In models with bacterial impact, NF-κB activation was associated with upregulation of inflammatory mediators (IL-1β [103], IL-6 [96,97,99], IL-10 [96], IL-18 [103], TNF-α [96,97,99,103], CXCL1 [97], CXCL2 [97]), fibrosis mediator (TGF-β1 [99,100]), macrophage recruitment (CCL1, CCL8 [96]), and fibrosis markers (α-SMA [100,103]). Although CXCL1 has been reported as an angiogenic factor in some studies [10,101], it was considered an inflammatory mediator in other [97]. Importantly, the absence of data for certain mediators and markers does not imply a lack of regulation but rather reflects the limited scope of measurements in current studies, which represents one of the limitations of the existing literature.

Considering further limitations of the reviewed studies, both peritoneal fibrosis and peritoneal adhesion models were induced in diverse ways, resulting in heterogeneous assessments of NF-κB activation [10,40,41,68,69,70,71,72,73,74,75,76,77,78,79,80,81,82,83,84,85,86,87,88,89,90,91,92,93,94,95,96,97,98,99,100,101,102,103], including investigations of specific pathways such as NFAT5/NF-κB [40,71], PKC/NF-κB [70], TLR4/MyD88/NF-κB [72], TAK1/NF-κB [74], and STAT3/NF-κB [78]. Therefore, the discussed results are not directly comparable across studies. Moreover, even among studies evaluating NF-κB-targeted therapies, there was a lack of homogeneity and reproducibility, so that each active compound was tested in a different model and using different protocols, thereby limiting validation of the findings. For example, although two studies examined inhibition of NF-κB activity by vitamin D analogs [89,98], one investigated peritoneal fibrosis in a non-CKD mouse model induced by chlorhexidine gluconate [89], whereas the other examined peritoneal adhesions in a rat uterine cauterization model [98]. The peritoneal fibrosis study shown that calcitriol reduces macrophage infiltration (F4/80) and upregulation of fibrosis (collagen III, α-SMA) and inflammatory markers (MCP-1) by inhibiting both the TGF-β/Smad pathway and NF-κB activity [89]. In contrast, the peritoneal adhesion study reported that cholecalciferol reduced injury-induced inflammation and adhesion formation—assessed only by histopathological scoring—through inhibition of NF-κB activity [98]. Although both studies reached similar conclusions, their experimental designs were not comparable. Likewise, two studies evaluated NF-κB activity in the context of novel biomaterials designed to prevent peritoneal adhesion formation [102,103]; however, one employed biocompatible anti-adhesion scaffolds [102], whereas the other used biomimetic enzymes with antioxidant and anti-inflammatory properties [103]. In addition, several studies reported inhibition of NF-κB activity by plant-derived active compounds [41,83,84,85,86,87,94,99,102]; yet each study investigated a different substance, including a natural NF-κB inhibitor from *Tanacetum balsamita* [41], a carotenoid [83], a glycoside from grapes and berries [84], a tea-derived polyphenol [85], a herbal extract and its major flavonoid [86], a lignan from *Arctium lappa* [87], a steroid saponin from *Dioscorea nipponica* Makino [94], a phenolic complex from *Rhus chinensis* [99], and lecithin and oregano essential oil used to coat anti-adhesion scaffolds [102]. Given this high heterogeneity of plant-derived compounds, the results of these studies are also not directly comparable. Despite high heterogeneity of studies on NF-κB-targeted therapy, both peritoneal fibrosis [41,81,82,83,84,85,86,87,88,89,90,91,92,93,94] and peritoneal adhesions [98,99,100,101,102,103] were significantly attenuated by inhibition of the PKC/NF-κB [82], TAK1/p38MAPK/NF-κB [86], and TLR4/NF-κB [93,94,100], ROS/NF-κB [83], NLRP3/NF-κB [84], DPP4/GLP-1R/NF-κB [92], and Piezo1/NF-κB [103] pathways, or by inhibiting components of NF-κB signaling, particularly through suppression activation of NF-κB [41,81,85,88,89,90,91,98,99,101] or phosphorylation of IκBα [87]. Highlighting the marked imbalance between studies on peritoneal fibrosis and peritoneal adhesions, it may be observed that future research on peritoneal adhesions could benefit not only from findings in peritoneal fibrosis research but also from adapting experimental protocols used in fibrosis models to intra-abdominal adhesion models. Finally, the current literature lacks human clinical trials evaluating any of the compounds proposed in NF-κB-targeted therapy studies for the prevention of peritoneal fibrosis or peritoneal adhesion formation. Therefore, the clinical relevance of these preclinical findings remains unclear and requires further investigation to avoid overgeneralization of the experimental findings. This underscores the limited translation of basic and preclinical findings into clinical practice.

However, while highlighting the limitations of the included studies, it is also important to acknowledge the limitations of this systematic review itself. One such limitation is that controlled vocabulary and broader Boolean strategies were not tested during the search strategy stage of this revision. Another limitation is that the search strategy focused specifically on “peritoneal fibrosis/adhesion” and “NF-κB,” which may have resulted in a relatively narrow scope. The search did not explicitly include NF-κB components such as p50, p65, RelA, IκB, IKK or the names of downstream mediators of the studied pathways. Nevertheless, expanded synonyms for NF-κB were used, under the assumption that at least the abstract of any study examining the NF-κB pathway would reference it directly. For this reason, the MeSH terms have not been extended to include individual NF-κB components. Expanding MeSH terms to include downstream pathway components was not feasible at the search planning stage, as these components are results identified in this study—the first systematic review of NF-κB’s role in peritoneal fibrosis and adhesions. Despite the considered limitations, some relevant publications may not have been captured, introducing the potential for retrieval bias due to underestimation.

Interestingly, among animal studies, only mouse and rat models—both in vivo and in vitro—were used in the reviewed studies. Consequently, no studies have investigated the role of NF-κB in the regulation, prevention, or therapy of peritoneal fibrosis or adhesion in companion or livestock animals. This is notable because, i.a., dogs [118,119], cats [118,119], and horses [120,121] suffer from CDK, and PD can be clinically applied as a renal replacement therapy in these species [122]. Similarly, it was reported that dogs [123], cats [124], and horses [125] develop peritoneal adhesions, particularly after abdominal surgery, which represent a major clinical and surgical problem and can lead to complications similar to those in human medicine [126], such as intestinal obstruction, abdominal pain, and potentially fatal outcomes. This gap in the existing knowledge highlights an opportunity to advance veterinary medicine by translating findings from laboratory animal models and human studies into veterinary clinical practice.

## 5. Conclusions

Control of NF-κB activity in the peritoneal mesothelium during PD and intra-abdominal surgical interventions may be beneficial in preventing dysfunction of the peritoneal membrane and post-inflammatory complications. Regulating NF-κB-mediated inflammatory responses in the peritoneum could help limit persistent inflammation before it triggers further morphological and functional alterations. However, the direct NF-κB signaling pathways and their potential therapeutic modulation still require further, more homogeneous investigation to clarify the clinical relevance of preclinical findings and to avoid overgeneralization of experimental findings, particularly since no clinical trials of NF-κB-targeted therapies are currently available in the literature. Both preclinical and future clinical studies warrant greater attention, especially in the context of peritoneal adhesion formation, which remains considerably less studied than peritoneal fibrosis.

## Figures and Tables

**Table 1:** Studies on NF-κB-mediated regulation of peritoneal fibrosis.

Paper	Treatment	In Vitro Model	Cell Stimulation	In Vivo Model	Fibrosis Induction	Molecular Markers	Molecular Methods	Outcome
Marchant et al., 2025 [10]	STING gene deletion or inhibitors	human peritoneal biopsies/human peritoneal MC culture	AMCM/LPS	CKD (5/6 nephrectomy) and non-CKD mouse models	CG solution/bacteria	Fibronectin, α-SMA, MCP-1, IL-1β, IL-6, TNF-α TGF-β1, VEGF, CXCL1, STING, NF-κB, IκBα, IκBε, IKKε *	RNA-seq, qPCR, WB, IF **, IHC **, FC **	STING genetic deletion and inhibition downregulates: predominately inflammatory markers by inhibiting NF-κB activation; MMT and fibrosis markers by blocking activated macrophages and inhibiting TGF-β1-driven signaling pathway; angiogenesis markers via independent mechanism
Kitterer et al., 2015 [40]	not applied	human peritoneal MC culture/human peritoneal biopsies	glucose/ effluent PD dialysate	not applied	not applied	MCP-1, CD68, NFAT5, NF-κB *	IHC, qPCR **	Glucose upregulates MCP-1 secretion by activating NFAT5/NF-κB pathway both in MC culture and biopsy promoting macrophages migration to peritoneum
Witowski et al., 2001 [68]	IL-1 inhibitor	human peritoneal MC culture	effluent PD dialysate or TNF-α/IL-1β	not applied	not applied	MCP-1, IL-8, NF-κB, IκBα *	qPCR, EMSA ELISA **	Peritoneal MCs are capable to secrete MCP-1 and IL-8 probably by activating NF-κB
Sosińska et al., 2016 [69]	NF-κB inhibitor	human peritoneal MC culture	effluent PD dialysate	not applied	not applied	MCP-1, IL-6, HAS 1-3	qPCR **, ELISA **	MCP-1, IL-6, and HAS secretion by peritoneal MCs is suppress by NF-κB inhibition
Lee et al., 2001 [70]	MCP-1, PTK AP-1/NF-κB inhibitor	human peritoneal MC culture	glucose/mannitol	not applied	not applied	MCP-1, AP-1, NF-κB *	EMSA, qPCR **, ELISA **	Glucose upregulates MCP-1 secretion by activating PTK/AP-1 pathway but not PKC/NF-κB pathway
Küper et al., 2012 [71]	NF-κB inhibitor	human peritoneal MC culture	glucose/mannitol	not applied	not applied	MCP-1, NFAT5, NF-κB, p-NF-κB *	WB, SEAP, qPCR **, ELISA **	High osmolality upregulates MCP-1 secretion by activating NFAT5/NF-κB pathway
Choi et al., 2017 [72]	not applied	human peritoneal MC culture	glucose/mannitol	not applied	not applied	Fibronectin, α-SMA, MCP-1, TGF-β1, TLR 1-6, MyD88, NF-κB *	WB, qPCR **, ELISA **	Glucose upregulates inflammatory and fibrosis markers by activating TLR4/MyD88/NF-κB pathway
Zhou et al., 2013 [73]	PPARγ activator/ AP-1 inhibitor/ NF-κB inhibitor	rat peritoneal MC culture	glucose	not applied	not applied	Fibronectin, collagen I, PAI-1, PPARγ, AP-1, NF-κB, IκBα *	WB, LUC, qPCR **, ELISA **	Glucose upregulates fibrosis markers and downregulates PPARγ expression. PPARγ activation downregulates fibrosis markers by inhibiting AP-1 and NF-κB activity
Su et al., 2014 [74]	PPARβ/δ activator	rat peritoneal MC culture	glucose	CKD (5/6 nephrectomy) rat model	glucose- based PD solution/LPS	MCP-1, IL-6, TNF-α, TGF-β1, TAK1, p-TAK1, p-NF-κB, IκBα, p-IκBα *	WB, IHC	Glucose upregulates inflammatory markers. PPARβ/δ activation downregulates inflammatory markers by inhibiting TAK1/NF-κB pathway
Luo et al., 2022 [75]	PGE_2_ receptor subtype 4 inhibitor	human peritoneal biopsies/ rat peritoneal MC culture	glucose/mannitol	non-CKD rat model	glucose- based PD solution	Vimentin, collagen I, fibronectin, MCP-1, IL-1β, TNF-α, EP4, NLRP3, NF-κB, p-NF-κB *	WB, IHC **, qPCR **, ELISA **	Glucose upregulates PGE_2_ receptor subtype 4 expression. PGE_2_ receptor subtype 4 inhibition downregulates MMT and fibrosis markers by inhibiting NLRP3 activation and inflammatory markers by suppressing NF-κB activation
Strippoli et al., 2012 [76]	TAK1 gene silencing	human peritoneal MC culture/human effluent- derived MC culture	TGF-β1/ IL-1β	not applied	not applied	E-cadherin, vimentin, fibronectin, PAI-1, TAK1, p-TAK1, Smad1-5, p-Smad3, p-c-Jun, Snail1, NF-κB *	LUC, WB **, IF **, PCR **	TAK1 inhibition downregulates MMT and fibrosis markers by reducing activity of Smads, AP-1, NF-κB, and Snail
Wang et al., 2017 [77]	Src inhibitor	human peritoneal MC culture	TGF-β1	non-CKD rat model	CG solution	Fibronectin, collagen I, α-SMA, MCP-1, IL-1β, IL-6, TNF-α, TGF-β1, p-Smad3, Smad3, p-Src, Src, p-NF-κB, NF-κB *	WB, IHC **, siRNA **, ELISA **	Src inhibition downregulates fibrosis markers by inhibiting TGF-β/Smad pathway and downregulates inflammatory markers by inhibiting NF-κB activation
Shi et al., 2021 [78]	Autophagy inhibitor	human peritoneal MC culture	TGF-β1	non-CKD rat model	glucose- based PD solution/ CG solution	E-cadherin, fibronectin, collagen I, MCP-1, IL-1β, IL-6, CD68, TGF-β1, TGF-βR1, Smad3, p-Smad3, p-STAT3, STAT3, NF-κB, p-NF-κB *	WB, IF, IHC, siRNA **, ELISA **	TGF-β1 stimulates autophagic activity. Blockade of autophagy prevents MMT and fibrosis markers upregulation by suppressing TGF-β1/Smad pathway and decreases inflammatory markers upregulation by suppressing STAT3/NF-κB crosstalk
Shi et al., 2020 [79]	EZH2 gene deletion or inhibitor	human peritoneal MC culture/human effluent/ human effluent- derived MC culture	TGF-β1	non-CKD mouse model	glucose- based PD solution/ CG solution	E-cadherin, collagen I, α-SMA, MCP-1, IL-1β, IL-6, TNF-α, TGF-β1, VEGF, EZH2, Smad3, TGF-βR1, p-STAT3, STAT3, p-NF-κB, NF-κB *	WB, IF **, IHC **, siRNA **, ELISA **	EZH2 genetic deletion and inhibition downregulates MMT and fibrosis markers by inhibiting TGF-β/Smad pathway and downregulates inflammatory and angiogenesis markers by suppressing STAT3 and NF-κB activation
Yu et al. 2021 [80]	Fut8- knockdown of EGFR	not applied	not applied	non-CKD rat model	glucose- based PD solution	Collagen I, MCP-1, Fut8, EGF, EGFR, p-STAT3, STAT3, p-NF-κB, NF-κB	WB, IHC, ELISA **	EGFR knockdown downregulates fibrosis and inflammatory markers by suppressing STAT3 and NF-κB activation

Footnotes: *—inter alia; **—applied in the study, but not to the NF-κB assessment; α-SMA—α-smooth muscle actin; AMCM—activated macrophage-conditioned media; AP-1—activator protein 1; CD68—cluster of differentiation 68 expressed by cells in the monocyte/macrophage lineage; CG—chlorhexidine gluconate; CKD—chronic kidney disease; CXCL1—chemokine (C-X-C motif) ligand 1; EGF—epidermal growth factor; EGFR—epidermal growth factor receptor; ELISA—enzyme-linked immunosorbent assay; EMSA—electrophoretic mobility shift assay; EP4—prostaglandin E_2_ receptor subtype 4; EZH2—Enhancer of zeste homolog 2; FC—flow cytometry; Fut8—α1,6-fucosyltransferase; IκB—inhibitor kappa B; HAS—hyaluronan synthase; IF—immunofluorescence staining; IHC—immunohistochemical staining; IKKε—IκB kinase ε; IL—interleukin; LPS—lipopolysaccharide; LUC—luciferase assay; MC—mesothelial cell; MCP-1—monocyte chemoattractant protein 1; MMT—mesothelial-to-mesenchymal transition; MyD88—myeloid differentiation primary response 88; NF-κB—nuclear factor kappa B; NFAT5—nuclear factor of activated T cells 5; NLRP3—NLR family pyrin domain containing 3; p-—phosphorylated; PAI-1—plasminogen activator inhibitor type 1; PD—peritoneal dialysis; PGE_2_—prostaglandin E_2_; PKC—protein kinase C; PPAR—peroxisome proliferator-activated receptor; PTK—protein tyrosine kinase; qPCR—quantitative real-time PCR; RNA-seq—RNA next-generation sequencing; SEAP—secreted alkaline phosphatase system; siRNA—siRNA knockdown; STAT3—signal transducer and activator of transcription 3; STING—stimulator of interferon genes; TAK1—TGF-β-activated kinase 1; TGF-β—transforming growth factor β; TGF-βR1—transforming growth factor β receptor 1; TLR—Toll-like receptor; TNF-α—tumor necrosis factor α; VEGF—vascular endothelial growth factor; WB—Western blot.

**Table 2:** Studies on NF-κB-targeted therapy for the prevention of peritoneal fibrosis.

Paper	Treatment	In Vitro Model	Cell Stimulation	In Vivo Model	Fibrosis Induction	Molecular Markers	Molecular Methods	Outcome
Zhang et al., 2022 [41]	Parthenolide (plant NF-κB inhibitor)	human peritoneal biopsies/ human and rat peritoneal MC culture	glucose/mannitol	non-CKD mouse model	glucose- based PD solution	E-cadherin, fibronectin, collagen I, α-SMA, MCP-1, IL-6, TNF-α, TGF-β1, Smad2/3, NF-κB, p-NF-κB, IκBα, p-IκBα *	WB, LUC, IF, qPCR ** ELISA **	Parthenolide suppresses glucose-induced MMT, fibrosis, and inflammatory markers upregulation by inhibiting TGF-β1/Smad pathway, NF-κB activation, and TGF-β/NF-κB crosstalk
Haslinger et al., 2003 [81]	Simvastatin (statin)	human peritoneal MC culture	TNF-α	not applied	not applied	t-PA, PAI-1, c-Jun, c-Fos, NF-κB	WB, LUC, ELISA **	Simvastatin enhances t-PA and suppresses PAI-1 synthesis by inhibiting AP-1 and NF-κB activity
Matsuo et al., 2006 [82]	Prednisolone (corticosteroid)	rat peritoneal MC culture	glucose/mannitol	not applied	not applied	MCP-1, GR, PKC, NF-κB, IκBα *	WB, qPCR ** ELISA **	Prednisolone suppresses glucose- and osmotic-induced MCP-1 secretion by inhibiting PKC/NF-κB pathway
Hara et al., 2017 [83]	Astaxanthin (plant carotenoid)	rat peritoneal MC culture	glucose	not applied	not applied	E-cadherin, α-SMA, TNF-α, TGF-β, VEGF, ROS, NF-κB *	IF, ELISA, qPCR **, ROS assay **	Astaxanthin prevents glucose-induced MMT, fibrosis, and inflammatory markers upregulation by inhibiting ROS/NF-κB pathway
Liu et al., 2025 [84]	Polydatin (plant glycoside)	human peritoneal MC culture	glucose/mannitol	non-CKD rat model	glucose- based PD solution	E-cadherin, collagen I, α-SMA, IL-1β, IL-18, TGF-β, VEGF, ROS, NLRP3, NF-κB, p-NF-κB *	WB, IHC **, IF **	Resveratrol glycoside mitigates glucose-induced MMT, fibrosis, angiogenesis, inflammatory markers upregulation and ROS production by inhibiting NLRP3/NF-κB pathway
Kitamura et al., 2012 [85]	EGCG (plant polyphenol)	not applied	not applied	non-CKD mouse model	glucose- based PD solution/glucose DP	MCP-1, TGF-β, VEGF, ROS, NF-κB *	SWH, IHC **	EGCG reduces glucose DP-induced angiogenesis and inflammatory markers and ROS production by inhibiting NF-κB activity
Tang et al., 2025 [86]	Apigenin (plant flavonoid)/SBD III (plant extract)	human peritoneal MC culture	glucose- based PD solution	CKD (5/6 nephrectomy) mouse model	glucose- based PD solution	E-cadherin, fibronectin, collagen I, α-SMA, TGF-β1, TAK1, p38MAPK, NF-κB	qPCR, WB, IF **	Apigenin and SBD III reduces glucose-induced MMT and fibrosis markers upregulation by inhibiting TAK1/p38MAPK/NF-κB pathway
Jin et al., 2019 [87]	Arctigenin (plant lignan)	human peritoneal MC culture	TGF-β1	not applied	not applied	E-cadherin, fibronectin, collagen I, α-SMA, PAI-1, AMPK, p-AMPK, NF-κB, p-NF-κB, IκBα, p-IκBα *	WB, LUC, IF, qPCR **, ELISA **	Arctigenin suppresses TGF-β-induced MMT and fibrosis markers by inhibiting IκBα phosphorylation and activating AMPK/NF-κB pathway
Shinkai et al., 2024 [88]	Pemafibrate (PPARα activator)	human peritoneal MC culture	IFN-γ	non-CKD mouse model **	glucose DP **	Fibronectin, IL-1β, IL-6, TNF-α, TGF-β1, p-c-Jun, NF-κB, p-NF-κB, p-IκBα *	WB, IHC **, ELISA **, qPCR **	Pemafibrate inhibits IFN-γ-induced MMT, fibrosis, and inflammatory markers upregulation by inhibiting AP-1 and NF-κB activity
Hirose et al., 2013 [89]	Calcitriol (vitamin D)	not applied	not applied	non-CKD mouse model	CG solution	Collagen III, α-SMA, MCP-1, TGF-β, F4/80, VDR, p-Smad2/3, NF-κB	SWH, IHC **	Calcitriol reduces CG-induced fibrosis and inflammatory markers upregulation and macrophage infiltration by inhibiting TGF-β/Smad pathway and NF-κB activation
Xiong et al., 2014 [90]	Suramin (growth factor inhibitor)	not applied	not applied	non-CKD rat model	CG solution	Fibronectin, collagen I, α-SMA, MCP-1, IL-1β, IL-6, TNF-α, TGF-β1, VEGF, CD68, p-Smad3, Smad3, NF-κB, p-NF-κB, IκBα, p-IκBα	WB, IHC **, ELISA **	Suramin reduces CG-induced fibrosis, inflammatory, and angiogenesis markers upregulation and macrophage infiltration by inhibiting TGF-β1/Smad pathway and NF-κB activation
Abe et al., 2016 [91]	Chondroitin sulfate (glycosaminoglycan)	not applied	not applied	non-CKD mouse model	CG solution	MCP-1, IL-1β, F4/80, p-Smad2/3, NF-κB, IκBα *	SWH, IF **, IHC **, ELISA **	Chondroitin sulfate suppresses CG- induced inflammatory markers upregulation and macrophage infiltration by inhibiting TGF-β/Smad pathway and NF-κB activation
Yang et al., 2025 [92]	Dulaglutide (GLP-1 receptor activator)	human pleural MC culture	uremic toxin/LPS	non-CKD and CKD (uremic toxin- induced) rat models	CG solution	Fibronectin, collagen I, α-SMA, TNF-α, TGF-β, p-Smad3, Smad3, ROS, DPP4, GLP-1, GLP-1R, Nrf2, TLR2, TLR4, NF-κB, p-NF-κB *	WB, FC **, siRNA **	Dulaglutide reduces CG-induced, uremic-induced, and LPS-induced fibrosis markers upregulation by inhibiting TGF-β/Smad pathway; oxidative stress by inhibiting DPP4/GLP-1R/Nrf2/ROS pathway; and inflammatory markers upregulation by DPP4/GLP-1R/NF-κB pathway and TGF-β/NF-κB crosstalk
Shi et al., 2016 [93]	Melatonin (amino acid derived hormone)	human peritoneal MC culture	LPS	not applied	not applied	E-cadherin, vimentin, α-SMA, TLR4, c-Jun, Snail, NF-κB *	qPCR, IF **, WB **	Melatonin suppresses LPS-induced MMT and fibrosis markers upregulation by inhibiting TLR4/AP-1 and TLR4/NF-κB/Snail pathways
Shao et al., 2019 [94]	Dioscin (plant steroid saponin)	human peritoneal MC culture	LPS	not applied	not applied	E-cadherin, vimentin, fibronectin, collagen I, α-SMA, IL-1β, IL-6, TNF-α, TGF-β1, p-Smad2, Smad2, TLR4, MyD88, NF-κB *	WB	Dioscin attenuates LPS-induced MMT, fibrosis, and inflammatory markers upregulation by inhibiting TGF-β1/Smad and TLR4/MyD88/NF-κB pathways

Footnotes: *—inter alia; **—applied in the study, but not to the NF-κB assessment; α-SMA—α-smooth muscle actin; AP-1—activator protein 1; AMPK—adenosine monophosphate-activated protein kinase; CD68—cluster of differentiation 68 expressed by cells in the monocyte/macrophage lineage; CG—chlorhexidine gluconate; CKD—chronic kidney disease; DP—degradation products; DPP4—dipeptidyl peptidase 4; EGCG—(−)-epigallocatechin-3-gallate; ELISA—enzyme-linked immunosorbent assay; F4/80—surface protein of mouse macrophage; FC—flow cytometry; GLP-1—glucagon-like peptide 1; GLP-1R—glucagon-like peptide 1 receptor; GR—glucocorticoid receptor; IκBα—inhibitor kappa B α; IF—immunofluorescence staining; IFN-γ—interferon-γ; IHC—immunohistochemical staining; IL—interleukin; LPS—lipopolysaccharide; LUC—luciferase assay; MAPK—mitogen-activated protein kinase; MC—mesothelial cell; MCP-1—monocyte chemoattractant protein 1; MMT—mesothelial-to-mesenchymal transition; MyD88—myeloid differentiation primary response 88; NF-κB—nuclear factor kappa B; NLRP3—NLR family pyrin domain containing 3; Nrf2—nuclear factor erythroid 2-related factor 2; p-—phosphorylated; p38—p38 mitogen-activated protein kinase; PAI-1—plasminogen activator inhibitor type 1; PD—peritoneal dialysis; PKC—protein kinase C; PPAR—peroxisome proliferator-activated receptor; qPCR—quantitative real-time PCR; ROS—reactive oxidative species; SBD III—Shenbing Decoction III; siRNA—siRNA knockdown; SWH—southwestern histochemistry; t-PA—tissue-type plasminogen activator; TAK1—TGF-β-activated kinase 1; TGF-β—transforming growth factor β; TLR—Toll-like receptor; TNF-α—tumor necrosis factor α; VDR—vitamin D receptor; VEGF—vascular endothelial growth factor; WB—Western blot.

**Table 3:** Studies on NF-κB-mediated regulation of peritoneal adhesions.

Paper	Treatment	In Vitro Model	Cell Stimulation	In Vivo Model	Adhesion Induction	Tissue and Molecular Markers	Molecular Methods	Outcome
Marchant et al., 2025 [10]	STING gene deletion or inhibitors	human peritoneal biopsies ***/ human peritoneal MC culture ***	AMCM ***/LPS ***	intra- abdominal adhesion mouse model	ischemic buttons	Adhesion score, MCP-1, RANTES, IP-10, IFIT1, USP18, Mx2, STING, TBK1, IRF3, p-IκBα *	RNA-seq, qPCR, WB, IF **, IHC **, FC **, HP **	STING genetic deletion and inhibition reduce inflammatory markers and interferon-induced proteins upregulation and adhesion formation by inhibiting TBK1/IRF3 pathway and NF-κB activation
Jiang et al., 2009 [95]	not applied	normal/ adhesion-derived human peritoneal fibroblast culture	hypoxia	not applied	not applied	iNOS, NF-κB, IκBα, p-IκBα	WB, qPCR	Normal and adhesion peritoneal MCs are capable of increasing iNOS expression by a hypoxia-induced mechanism involving NF-κB activation
Oshio et al., 2014 [96]	CCR8 gene deletion or inhibitor/NF-κB inhibitor *	mouse peritoneal macrophage culture	LPS	three intra- abdominal adhesion mouse models	cauterization of cecum/ abrasion of cecum/ ischemic buttons	Adhesion score, IL-6, IL-10, TNF-α, CCL1, CCL8, TLR4, ERK, JNK, p38, p-JNK, p-c-Jun, p-IκBα *	PA, IF **, qPCR **, ELISA **, HP **	CCR8 inhibition decreases LPS- induced inflammatory markers upregulation and macrophage migration as well as injury-induced adhesion formation by inhibiting TLR4/MAPK/AP-1 and TLR4/NF-κB pathways
Bian et al., 2020 [97]	not applied	not applied	not applied	intra- abdominal adhesion rat model	cauterization of cecum	Adhesion score, IL-6, TNF-α, CXCL1, CXCL2, TLR4, MyD88, NF-κB *	DEGs identification, WB, qPCR **, HP **	Peritoneal adhesion formation is stimulated by inflammatory markers upregulation by activating TLR4/MyD88/NF-κB pathway

Footnotes: *—inter alia; **—applied in the study, but not to the NF-κB assessment; ***—applied in the study, but not to the adhesion assessment; AMCM—activated macrophage-conditioned media; AP-1—activator protein 1; CCL—chemokine (C-C motif) ligand; CXCL—chemokine (C-X-C motif) ligand; CCR8—chemokine (C-C motif) receptor 8; DEGs—differentially expressed genes; ELISA—enzyme-linked immunosorbent assay; ERK—extracellular signal-regulated kinase; FC—flow cytometry; HP—histopathologic examination; IκBα—inhibitor kappa B α; IF—immunofluorescence staining; IFIT1—interferon-induced protein with tetratricopeptide repeats 1; IHC—immunohistochemical staining; IL—interleukin; iNOS—inducible nitric oxide synthase; IP-10—interferon gamma-induced protein 10; IRF3—interferon regulatory factor 3; JNK—c-Jun N-terminal kinase; LPS—lipopolysaccharide; MAPK—mitogen-activated protein kinase; MC—mesothelial cell; MCP-1—monocyte chemoattractant protein 1; MyD88—myeloid differentiation primary response 88; NF-κB—nuclear factor kappa B; p-—phosphorylated; p38—p38 mitogen-activated protein kinase; PA—phosphoprotein assays; qPCR—quantitative real-time PCR; RNA-seq—RNA next-generation sequencing; STING—stimulator of interferon genes; TBK1—TANK-binding kinase 1; TLR4—Toll-like receptor 4; TNF-α—tumor necrosis factor α; USP18—ubiquitin-specific protease 18; WB—Western blot.

**Table 4:** Studies on NF-κB-targeted therapy for the prevention of peritoneal adhesions.

Paper	Treatment	In Vitro Model	Cell Stimulation	In Vivo Model	Adhesion Induction	Tissue and Molecular Markers	Molecular Methods	Outcome
Sahbaz et al., 2015 [98]	Chole- calciferol (vitamin D)	not applied	not applied	intra- abdominal adhesion rat model	cauterization of uterus	Adhesion score, inflammation score, NF-κB	IHC, HP **	Cholecalciferol reduces injury-induced inflammation and adhesion formation by inhibiting NF-κB activity
Wei et al., 2018 [99]	Gallic acid (plant phenolic complex)	not applied	not applied	intra- abdominal adhesion rat model	abrasion of cecum and parietal peritoneum	Adhesion score, IL-6, TNF-α, TGF-β, NF-κB, p-NF-κB	WB, IHC **, ELISA **, HP **	Gallic acid reduces injury-induced inflammatory markers upregulation and adhesion formation by inhibiting NF-κB activation
Abbas et al., 2022 [100]	Androstenediol (steroid)	not applied	not applied	intra- abdominal adhesion rat model	abrasion of cecum	Adhesion score, α-SMA, MAD, SOD, HMGB1, TGF-1β, TLR4, NF-κB	ELISA	Androstenediol reduces injury- induced inflammatory and fibrosis markers upregulation, oxidative stress, and adhesion formation by inhibiting TLR4/NF-κB pathway
De Lazari et al., 2022 [101]	Sodium butyrate (fatty acid)	not applied	not applied	intra- abdominal adhesion mouse model	abdominal implant	Vascular score, MCP-1, TNF-α, TGF-1β, VEGF, CXCL1, NF-κB	WB, ELISA **, HP **	Sodium butyrate reduces injury-induced inflammatory and angiogenesis markers upregulation by inhibiting NF-κB activity
Wu et al., 2024 [102]	PLGA with plant oils (anti- adhesion membrane)	mouse macrophage culture ***	LPS ***	intra- abdominal adhesion rat model	injuring of peritoneum with a point hemorrhage	Adhesion score, collagen I, collagen III, α-SMA, p-Nrf2, p-NF-κB	WB, HP **	PLGA-based membrane reduces injury-induced fibrosis markers upregulation and adhesion formation by activating Nrf2 phosphorylation and inhibiting NF-κB activation
Yang et al., 2025 [103]	L-CMH/CD (hydrogel enzyme)	mouse peritoneal fibroblast culture	LPS/H_2_O_2_	intra- abdominal adhesion mouse model	abrasion of cecum and parietal peritoneum	Adhesion score, α-SMA, IL-1β, IL-18, TNF-α, ROS, Piezo1, NF-κB, p-NF-κB *	RNA-seq, qPCR, WB, IF **, HP **, ROS assay **	L-CMH/CD reduces LPS- and injury-induced inflammatory and fibrosis markers upregulation, oxidative stress, and adhesion formation by inhibiting Piezo1/NF-κB pathway

Footnotes: *—inter alia; **—applied in the study, but not to the NF-κB assessment; ***—applied in the study, but not to the adhesion assessment; α-SMA—α-smooth muscle actin; CXCL1—chemokine (C-X-C motif) ligand 1; ELISA—enzyme-linked immunosorbent assay; HMGB1—high mobility group box 1; HP—histopathologic examination; IF—immunofluorescence staining; IHC—immunohistochemical staining; IL—interleukin; L-CMH/CD—hydrogel microsphere-encapsulated SOD enzyme; LPS—lipopolysaccharide; MAD—malondialdehyde; MCP-1—monocyte chemoattractant protein 1; NF-κB—nuclear factor kappa B; Nrf2—nuclear factor erythroid 2-related factor 2; p-—phosphorylated; Piezo1—piezo-type mechanosensitive ion channel component 1; PLGA—poly(lactic-co-glycolic acid); qPCR—quantitative real-time PCR; RNA-seq—RNA next-generation sequencing; ROS—reactive oxidative species; SOD—superoxide dismutase; TLR4—Toll-like receptor 4; TNF-α—tumor necrosis factor α; TGF-β—transforming growth factor β; VEGF—vascular endothelial growth factor; WB—Western blot.

**Table 5:** Human and animal studies on peritoneal fibrosis.

Species	In Vitro Studies		In Vivo Studies	
	NF-κB-Mediated Regulation	NF-κB-Targeted Therapy	NF-κB-Mediated Regulation	NF-κB-Targeted Therapy
Human	peritoneal MC cultures [10,40,68,69,70,71,72,76,77,78,79]/ peritoneal biopsies [10,40,75]/ effluent-derived MC cultures [76,79]	peritoneal MC cultures [41,81,84,86,87,88,93,94]/ peritoneal biopsies [41]/ pleural MC cultures [92]	not applied	not applied
Mouse	not applied	not applied	non-CKD [10,79]/ CKD (5/6 nephrectomy) [10]	non-CKD [41,85,89,91]/ CKD (5/6 nephrectomy) [85]
Rat	peritoneal MC cultures [73,74,75]	peritoneal MC cultures [41,82,83]	non-CKD [75,77,78,80]/ CKD (5/6 nephrectomy) [74]	non-CKD [84,90,92] CKD (uremic toxin) [92]

Footnotes: CKD—chronic kidney disease; NF-κB—nuclear factor kappa B; MC—mesothelial cell.

**Table 6:** Human and animal studies on peritoneal adhesions.

Species	In Vitro Studies		In Vivo Studies	
	NF-κB-Mediated Regulation	NF-κB-Targeted Therapy	NF-κB-Mediated Regulation	NF-κB-Targeted Therapy
Human	adhesion-derived peritoneal fibroblast cultures [95]/ peritoneal fibroblast cultures [95]	not applied	not applied	not applied
Mouse	effluent-derived macrophage cultures [96]	peritoneal fibroblast cultures [102]	ischemic buttons [10,96]/ abrasion of cecum [96]/ cauterization of cecum [96]	abdominal implant [101]/ abrasion of cecum [103]
Rat	not applied	not applied	cauterization of cecum [97]	cauterization of uterus [98]/abrasion of cecum [99,100]/injuring of peritoneum with a point hemorrhage [102]

Footnotes: CKD—chronic kidney disease; NF-κB—nuclear factor kappa B; MC—mesothelial cell.

## Data Availability

The data presented in this study are available on request from the corresponding author.

## Supplementary Materials

The following supporting information can be downloaded at: https://www.mdpi.com/article/10.3390/ijms27052199/s1.
