# Supplementary material for: The Role of NF-κB in Peritoneal Fibrosis and Adhesion in Humans and Animals: A Systematic Review
Source: Int J Mol Sci. 2026 Feb 26;27(5):2199. doi: 10.3390/ijms27052199 (PMC12984160; doi:10.3390/ijms27052199)
Supplement: Supplementary file 1 [file ijms-27-02199-s001.zip › Supplementary file 1_NF-kB_24 01 2026.pdf]

Table S1: An example of data extraction template.

**General review information**	
**Name of review author completing this form:**	T.J.
**Date form completed:**	12.12.2025
**Name of review author checking the data extracted to this form:**	M.D.
**Author contact details for study**	Marta Ruiz-Ortega;<br>Cellular and Molecular Biology in Renal and Vascular Pathology Laboratory, Health Research Institute-Fundacion Jiménez Díaz University Hospital, Universidad Autonoma de Madrid (IIS-FJD, UAM), Av. Reyes Cataolicos 2, 28040 Madrid, Spain<br>e-mail: marta.ruiz.ortega@uam.es
**Further information required**	No
**Correspondence with authors successful or not; what information was received and when**	n/a
**Additional unpublished data?**	n/a
**Year of publication**	2025
**Record code**	Marchant 2025 STING inhibition alleviates experimental peritoneal damage: potential therapeutic relevance for peritoneal dialysis
**Notes**	
**Methods of the study**	
**Aim of study**	To report transcriptomic analysis in a preclinical model of peritoneal injury, which identified novel mediators involved in peritoneal damage and highlighted the cytosolic DNA-sensing pathway as one of the most relevant signaling pathways involved in peritoneal damage.
**Type of study**	1) experimental study<br>1.1) randomized controlled trial<br>1.2) non-randomized trial (prospective)

	1.2.1) cross-sectional study / 1.2.2) <u>cohort study</u> / 2.1.3) case-control study<br>2) observational study<br>2.1) analytic study (retrospective/prospective)<br>2.1.1) cross-sectional study / 2.1.2) cohort study / 2.1.3) case-control study<br>2.2) descriptive study (retrospective/prospective)<br>2.2.1) cross-sectional study / 2.2.2) case series / 2.2.3) case reports / 2.2.4) anatomical study<br>3) other (case reports / systematic review / narrative review / commentary / books)
**Item of study**	peritoneal fibrosis: <u>yes</u>/no; peritoneal adhesions: <u>yes</u>/no; NF-κB: <u>yes</u>/no
**Model used**	tissue and cell models: <u>yes</u>/no; animal model <u>yes</u>/no
**Peritoneal fibrosis section**	
***Ex vivo* model details**	type: human peritoneal biopsies / human peritoneal MC culture<br>cell stimulation: AMCM / LPS
***In vivo* model details**	type: CKD (5/6 nephrectomy) and non-CKD mouse models<br>fibrosis induction: CG solution / bacteria
**Treatment**	STING gene deletion or inhibitors
**Main molecular markers**	TBK1, IFNR1, IFNR2, IRF7, NF-κB, IκBα, IκBε, IKKε*
**Main molecular methods**	RNA-seq, qPCR, WB, IF**, IHC**, FC**
**Outcome description**	STING genetic deletion and inhibition reduce ETM by blocking activated macrophages and inflammation by inhibiting TBK1/IFN and IKKε/NF-κB pathways
**Peritoneal adhesions section**	
***Ex vivo* model details**	type: n/a<br>cell stimulation: n/a
***In vivo* model details**	type: intra-abdominal adhesion mouse model<br>adhesion induction: ischemic buttons
**Treatment**	STING gene deletion or inhibitors
**Main molecular markers**	TBK1, IFNR1, IFNR2, IRF7, NF-κB, IκBα, IκBε, IKKε*
**Main molecular methods**	RNA-seq, qPCR, WB, IF**, IHC**, FC**
**Outcome description**	STING genetic deletion and inhibition decrease macrophage recruitment, inflammation, and adhesions by inhibiting TBK1/IFN and IKKε/NF-κB pathways
**Funding source**	<u>Yes</u>/No/Unclear;<br>declaration: This study was funded by the Instituto de SaludCarlos III (ISCIII) through the projects PI20/00140, PI21/01453, PI23/00394, and RICORS2040-RD21/0005/0002, RD21/0005/0018, RD24/0004/0021, and

	RD24/0004/0001. This study was cofunded by the European Union-NextGenerationEU,Mecanismo para la recuperacion y la resiliencia (MRR). The grant IMPROVE-PD from the European Union's Horizon 2020 research and innovation program, under the Marie Skłodowska-Curie grant 812699, to ML-C, VM and MR-O. INNOREN 'P2022/BMD-7221: Nuevas estrategias diagnosticas y terapéuticas en enfermedad renal cronica' to VM and MR-O. Miguel Servet CP23/00025 to SR-M.
**Ethical approval**	Yes/No/Unclear;<br>declaration: All animal procedures were performed in mice according to the European Community and Animal Research: Reporting of In Vivo Experiments (ARRIVE) reporting guidelines for the care and use of laboratory animals, with the prior approval by the Animal Ethics Committee of the IIS-Fundacion Jiménez Díaz and Comunidad Autonoma de Madrid, Spain (PROEX 242.2/21). Experiments on peritoneal biopsies from patients were performed according to the Declaration of Helsinki guidelines. Written informed consent was obtained from all patients prior to sample obtention and approved by the Ethics Committee of Hospital Universitario La Paz, Madrid, Spain (HULP PI-4600; Ref. 07/253477.9/21).

Footnotes: n/a, not applicable; * - inter alia; ** - applied in the study, but not to the NF-κB assessment.
