# Supplementary material for: The Role of NF-κB in Peritoneal Fibrosis and Adhesion in Humans and Animals: A Systematic Review
Source: Int J Mol Sci. 2026 Feb 26;27(5):2199. doi: 10.3390/ijms27052199 (PMC12984160; doi:10.3390/ijms27052199)
Supplement: Supplementary file 1 [file ijms-27-02199-s001.zip › Supplementary file 2_NF-kB_20 02 2026.pdf]

Table S2: List of records excluded during full text screening.

No	Record	Reason of exclusion
1	Mondello, S.; Mazzon, E.; Di Paola, R.; Crisafulli, C.; Mondello, P.; Buemi, M.; et al. Thalidomide suppresses sclerosing encapsulating peritonitis in a rat experimental model. *Shock* **2009**, *32*, 332-339.	not about peritoneal fibrosis or adhesions
2	Wu, J.; Yang, X.; Zhang, Y.F.; Zhou, S.F.; Zhang, R.; Dong, X.Q.; et al. Angiotensin II upregulates Toll-like receptor 4 and enhances lipopolysaccharide-induced CD40 expression in rat peritoneal mesothelial cells. *Inflamm. Res.* **2009**, *58*, 473-482.	not about peritoneal fibrosis or adhesions
3	Morinelli, T.A.; Luttrell, L.M.; Strungs, E.G.; Ullian, M.E. Angiotensin II receptors and peritoneal dialysis-induced peritoneal fibrosis. *Int. J. Biochem. Cell Biol.* **2016**, *77*, 240-250.	narrative reviews
4	Artunc, F.; Lang, F. Mineralocorticoid and SGK1-sensitive inflammation and tissue fibrosis. *Nephron Physiol.* **2014**, *128*, 35-39.	narrative reviews
5	Aliakbarian, M.; Khodashahi, R.; Tavakkoli, M.; Ashrafzadeh, K.; Rahimi, H.; Khaleghi, E.; et al. Review the Role of Metabolism Reprogramming in the Pathogenesis of Post-surgical Adhesion: A New Therapeutic Strategy. *Curr. Top. Med. Chem.* **2023**, *23*, 2527-2534.	narrative reviews
6	Liu, H.; Xu, H.; Sun, H.; Xu, H.; Han, J.; Zhao, L.; et al. Tetrahydroberberrubine prevents peritoneal adhesion by suppressing inflammation and extracellular matrix accumulation. *Eur. J. Pharmacol.* **2023**, *954*, 175803.	full text not available
